# Prevalence of postpartum depression in the COVID-19 pandemic and associated factors: systematic review and meta-analysis

**DOI:** 10.1186/s12884-025-08262-z

**Published:** 2026-01-20

**Authors:** Marco Aurélio Knippel Galletta, Adriana Sayuri Hashimoto, Gabriel de Almeida Estrambk, Isabela Pinto Soares Verardo, Maria Helena Istake Cantagalli, Stela Verzinhasse Peres, Rossana Pulcineli Vieira Francisco

**Affiliations:** 1 Disciplina de Obstetricia, Departamento de Obstetricia E Ginecologia, Faculdade de Medicina FMUSP, Universidade de São Paulo, Avenida Doutor Enéas de Carvalho Aguiar 155, São Paulo, SP 05403-000 Brazil; 2 Hospital das Clinicas HCFMUSP, Faculdade de Medicina, Universidade de Sao Paulo, São Paulo, BR Brazil

**Keywords:** COVID-19, Postpartum depression, Pandemic, Prevalence, Risk factor

## Abstract

**Background:**

The COVID-19 pandemic created a disruptive scenario with an increase in the prevalence of postpartum depression (PPD) and new associated risk factors, which deserve to be better studied, in different global contexts, which led to the present systematic review study.

**Methods:**

Observational studies published in English, Portuguese, and Spanish between 2020 and 2025 were included, and a meta-analysis was conducted using a random-effects model.

**Results:**

An initial survey of 1741 articles, of which 90 studies were selected with a total of 64,6994 women evaluated for PPD, with a range between 50 (1) and 5,134 (2) women. The overall prevalence of postpartum depression during the COVID-19 pandemic was 28.48% (25.14—31.94), with rates of 23.52% (18.961—28.40) in studies that used the Edinburgh Postnatal Depression Scale (EPDS) as a diagnostic instrument with a cutoff point ≥ 13. Studies from 31 countries were included, with higher prevalence observed in Latin America (34.08%), with lower rates in Europe (31.50%), the Middle East (29.31%), USA/Canada (24.26%), and Asia (22.32%). There was a higher prevalence of PPD in countries with a lower Human Development Index (HDI) (30.95%), with higher COVID-19 CFR (32.56%), higher maternal mortality (30.43%); and with the highest Gender Inequality Index (GII) (35.41%). PPD rates increased with postpartum time, varying between 18.31% (up to 1 month), 20.78% (up to 3 months), 34.67% (up to 6 months) and 36.55% (up to 12 months). Additionally, 11 protective factors and 53 risk factors were identified, most related to the pandemic, but also with the presence of factors already consolidated in the literature before the pandemic.

**Discussion:**

There was a global increase in the prevalence of PPD during the pandemic, with an intensification of pre-existing regional differences, causing the impact of the pandemic to be different according to the region.

**Conclusions:**

The social and health crisis of the pandemic negatively impacted postpartum mental health, with significant regional differences.

**Trial registration:**

The study was registered in PROSPERO with the code CRD42023392973.

**Supplementary Information:**

The online version contains supplementary material available at 10.1186/s12884-025-08262-z.

## Background

Postpartum depression (PPD) is a mood disorder that may occur within the first year after childbirth, although diagnostic systems adopt narrower onset periods. The *Diagnostic and Statistical Manual of Mental Disorders*, Fifth Edition (DSM-5) specifies that a major depressive episode beginning during pregnancy or within 4 weeks after delivery qualifies for the specifier “with peripartum onset” [1], whereas the *International Classification of Diseases*, 11th Revision (ICD-11) allows for onset within 6 weeks postpartum [2]. Neither DSM-5-TR nor ICD-11 classifies PPD as a distinct entity, categorizing it under perinatal depression. In contrast, many clinical and research settings adopt broader criteria, often extending the onset window up to 12 months after childbirth [3, 4]. These discrepancies affect case identification, comparability of studies, and the design of targeted interventions, especially given evidence PPD may have unique clinical features and risk factors compared to depression during pregnancy or outside the perinatal period [5, 6].

PPD is the most common mental health condition in the perinatal period [7], affecting 10–15% of women in developed countries [8] and about 20.7% in developing countries [9]. The consequences of PPD extend beyond maternal psychological distress, posing significant risks to health and well-being of mothers and infants. For mothers, perinatal depression has been associated with increased all-cause mortality and a heightened risk of death from unnatural causes, including suicide [10]. In addition, depressive symptoms can impair the mother–infant bond, adversely affecting the child’s emotional regulation [11, 12], cognitive development [13, 14], and social functioning [15], and are linked to early breastfeeding cessation [16]. These adverse outcomes highlight the substantial public health burden imposed by PPD and emphasize the importance of early identification and intervention.

The diagnosis of PPD is formally established using DSM-5 and ICD-11 criteria, typically through structured clinical interviews [1, 2]; In clinical practice and research, however, screening tools are more frequently employed. Variability in the choice of instrument, timing of administration, and cut-off thresholds contributes to inconsistent case identification. The Edinburgh Postnatal Depression Scale (EPDS) is designed specifically for the perinatal period, excluding somatic symptoms common during postpartum recovery, which may improve specificity [17]. In contrast, the Patient Health Questionnaire-9 (PHQ-9) is a general depression measure with broad validity and comparability across populations, but in postpartum contexts it may be more susceptible to false positives due to the inclusion of somatic items [18]. In addition, diagnostic accuracy is further challenged by overlap between depressive symptoms and normal puerperal experiences: overdiagnosis may occur when typical postpartum changes (e.g., sleep disturbance, fatigue) are misclassified as depression, while underdiagnosis may occur when genuine depressive symptoms are dismissed as part of normal adjustment [19, 20]. Such misclassification can impair clinical care and produce heterogeneous research samples, limiting the validity of findings.

Prior to the COVID-19 pandemic, meta-analyses and large cohort studies consistently identified established risk factors for PPD, including a personal history of psychiatric illness [21], antenatal depressive or anxiety symptoms [22], low socioeconomic status [21], unintended pregnancy [23], limited social support [24], and exposure to major stressful life events[21]. During the pandemic, additional specific stressors emerged as potential novel risk factors for PPD. These include Maternal COVID-19 infection [25, 26] and Concern generated by pandemic [25, 27, 28].

Sociodemographic determinants, particularly the Human Development Index (HDI) and the Gender Inequality Index (GII), influence maternal mental health.. Low-HDI countries, often marked by weaker health systems and poorer care quality [29], faced greater challenges during the pandemic due to service overload and reduced access to essential care. The association of PPD with GII may reflect established risk factors, such as intimate partner violence [30], poor partner or marital relationship quality [31], and limited social support [32]. Furthermore, epidemiological indicators— maternal mortality and COVID-19 case fatality rate—may also shape the prevalence of PPD by reflecting systemic healthcare weaknesses and poorer care quality [33, 34].

Despite increasing research attention, important gaps remain regarding PPD during the COVID-19 pandemic. Although several systematic reviews have reported a significant rise in PPD prevalence during the pandemic [35, 36], findings are not universally consistent [37] and the magnitude of the increase varied across regions, populations, and study methodologies [35]. There is also ongoing debate about whether this increase reflects pandemic-related risk factors or an intensification of pre-existing ones..,Moreover, the influence of regional sociodemographic and epidemiological indicators (HDI, GII, maternal mortality and COVID-19 case fatality rate) on PPD prevalence remains insufficiently explored.

This study aims to systematically review the global prevalence of PPD during the COVID-19 pandemic, with subgroup analyses by screening instrument, geographic region, sociodemographic indicators (HDI, GII), and COVID-19-related mortality. It also seeks to identify risk factors associated with PPD in this context. This comprehensive approach is intended to clarify the pandemic’s impact on postpartum mental health and to enhance the interpretability of findings for the post-pandemic period.

## Methods

### Study protocol and study selection

This systematic review with meta-analysis follows the PRISMA (Preferred Reporting Items for Systematic Reviews and Meta-Analyses) guidelines [38]. The studies analyzed were independently selected by three reviewers (GAE, IPSV and MHIC). Disagreements regarding the inclusion or exclusion of any study were resolved through discussions with a fourth reviewer (MAKG) until consensus was reached. This systematic review is registered in PROSPERO under the code CRD42023392973.

### Search strategy

The researchers conducted a systematic review of the electronic databases Virtual Health Library (VHL), Pubmed, Scopus, Web of Science, and Embase. VHL is a database that publishes Latin-American and Caribbean literature. Full studies published between January 2020 and June 2025, were identified. Only studies in English, Portuguese, and Spanish were included. The search strategy included the following descriptors:

- **Postpartum depression–related terms:**
  - *○ “Depression, Postpartum”*
  - *○ “Depression, Post-Natal”*
  - *○ “Depression, Post-Partum”*
  - *○ “Depression, Postnatal”*
  - *○ “Post Natal Depression”*
  - *○ “Post Partum Depression”*
  - *○ “Post-Natal Depression”*
  - *○ “Post-Partum Depression”*
  - *○ “Postnatal Depression”*
  - *○ “Postpartum Depression”*
- **COVID-19–related terms:**
  - *○ “COVID-19”*
  - *○ “COVID19”*
  - *○ “COVID 19”*
  - *○ “SARS CoV 2”*
  - *○ “SARS Coronavirus 2”*
  - *○ “SARS-CoV-2”*
- **Final combination applied:**
  - ○ (**Postpartum depression–related terms**) **AND** (**COVID-19–related terms**)

### Inclusion and exclusion criteria

The inclusion criterion was studies providing complete data to assess the prevalence of postpartum depression up to one year postpartum.

The exclusion criteria were as follows:

- studies in which participants were assessed before the completion of one week postpartum;
• studies with small case series (< 50 women);
• studies without detailing the instrument used;
• studies conducted in specific populations,
• studies with intervention;
• systematic reviews and case reports.

### Data collection

The selection of papers was made independently by three researchers (GAE, IPSV and MHIC) using the Rayyan platform [39]. First, the articles were selected by excluding duplicates. Then, using the same platform, the screened studies were reviewed based on their titles and abstracts. At this stage, any disagreements were resolved by a fourth reviewer, a senior researcher. In the final stage, some articles were excluded after a full reading, with the agreement of the senior researcher.

The data of interest were organized using a standardized spreadsheet in the Excel-MS platform. Data extraction was performed by three reviewers (GAE, IPSV, and MHIC). Data from each article were extracted by a single reviewer, who extracted the total number of female participants in the study and the total number of affected women, according to the cutoff point established by each study for the scale used. Based on these data, the prevalence was calculated. Studies with fewer than 50 participants were excluded.

The information sought also included: instruments used in the evaluation with the respective cut-off score considered by each study, country of origin of the women, time elapsed since childbirth, type of study, and, when available, risk factors and associated protective factors.

Moreover, regional data analysis proved ineffective for a deeper understanding of the findings, considering that, when analyzing countries by continent, there were significant internal variations within each region, which suggests that generalizations may not accurately reflect the diversity of each nation. In this context, data from each country were collected on the following aspects: maternal mortality, number of COVID-19 cases, COVID-19 case fatality rate (CFR), gender inequality index (GII), and human development index (HDI). This type of more detailed analysis allowed for a more precise understanding of how the characteristics reflected by this data relate to the development of PPD in each country. Moreover, the relationship between structural gender inequality and a population's propensity to develop PPD [40], as well as the relationship between socioeconomic factors and PPD [41], had already been suggested in literature studies.

Maternal mortality rates for each country, the number of COVID-19 cases, and associated case-fatality rates were obtained from World Health Organization (WHO) reports. The Gender Inequality Index and the Human Development Index were sourced from United Nations Development Programme (UNDP) reports [42].

The maternal mortality ratio is calculated by dividing the number of maternal deaths by the number of live births in a specific geographical area and time period, multiplied by 100,000. This indicator serves as a proxy for both access to and the quality of the health system, given that most maternal deaths are preventable through comprehensive prenatal care, skilled assistance during childbirth and the postpartum period, adequate hospital infrastructure, and the availability of qualified health professionals. Therefore, it is understood that when a country has a high maternal mortality rate, there is a failure in the health system and there is no guarantee of women's basic health rights. For the meta-analysis, the maternal mortality ratio was divided into three groups: group 1 with a maternal mortality ratio of less than 10 deaths per 100,000 live births; group 2 with a ratio between 10 and 50 deaths; and group 3 with more than 50 deaths per 100,000 live births.

The Gender Inequality Index is a composite measure developed by the United Nations Development Programme (UNDP) to highlight gender disparities in three key dimensions of human development: reproductive health, empowerment and economic participation. By synthesizing multiple indicators into a single value, the GII not only allows us to quantify the degree of inequality between men and women, but also to compare this inequality across countries and over time.

The Gender Inequality Index (GII) ranges from 0 to 1, where a value of 0 denotes complete gender equality and a value of 1 represents the highest degree of inequality. Higher GII values, approaching 1, indicate social contexts in which women encounter significant barriers to accessing healthcare, education, paid employment, and political participation. The calculation of the GII is based on the aggregation of five indicators, organized into three key dimensions: reproductive health, empowerment (political representation and educational attainment), and labor market participation.

The COVID-19 case fatality rate was calculated by the ratio between the number of deaths and the number of confirmed cases of COVID-19 in the same period in the country, multiplied by 100. The data collected cover deaths from the beginning of the pandemic until August 2023 [43]. The decision to collect these data was based on the hypothesis that the difficulties faced by national health systems in managing the pandemic—reflected in higher mortality rates among confirmed cases—could have impacted women’s mental health to the extent of increasing the prevalence of PPD.

The Human Development Index is an index that allows comparison of the level of socioeconomic development between different countries and regions. The aspects analyzed by this index are: health, education and per capita income. Thus, an HDI threshold above 0.8 is generally considered very high development; between 0.70 and 0.79, high development; between 0.55 and 0.69, medium development; between 0.5 and 0.55, low development; and below 0.5, very low development. The HDI data used in this meta-analysis were extracted from the United Nations Human Development Report (2021–2022) [42].

### Quality assessment

The quality of the studies was assessed according to the characterization of the sample, the specification of the instrument employed and its cut-off point, the reporting of both the total number of participants and the number of affected cases, as well as the identification of the study design (cross-sectional or longitudinal). Studies with unclear or inconsistent methodology were excluded.

Also, the quality of the included studies was also assessed by four reviewers (ASH, GAE, IPSV, and MHIC) using the Joanna Briggs Institute Critical Appraisal Checklist for Analytical Cross-Sectional Studies [44] (Supplementary Table 1). Each study was assessed by only one reviewer. This scale considers whether the inclusion criteria for the sample in each article are well-defined, if there is a detailed description of the study participants, if the exposure was measured in a valid and reliable manner, if the measurement of the condition was conducted appropriately, if confounding factors were identified, if the handling of confounding factors was described, if the outcome was measured in a valid and reliable way, and if the statistical analysis used was appropriate. Following the assessment using this scale, none of the articles were excluded.

### Statistical analysis

The pooled prevalence and 95% confidence intervals (CI) of depression were calculated using the DerSimonian–Laird random-effects model. To stabilize the variances, a Freeman–Tukey double arcsine transformation was applied. The heterogeneity was calculated using Cochran’s Q test and the I^2^ statistic, which quantifies the proportion of total variability attributed to differences between studies. A *p* value greater than 0.05 in Cochranan’s Q test indicates the absence of significant heterogeneity. I^2^ values were classified as follows: insignificant (0–25%), low (26–50%), moderate (51–75%) and high heterogeneity (> 75%). In addition, subgroup analysis was conducted to examine the influence of subgroups (instrument used, cut-off point, study type, postpartum time elapsed, and geographical region where the study was conducted, as well as socioeconomic and maternal and COVID mortality characteristics in such countries) on the overall prevalence estimate by recalculating the pooled estimates for the remainder of the studies after removing one study at a time. Differences between subgroups were evaluated using the Q_between statistic, which compares the amount of heterogeneity explained by subgroup classification relative to the overall heterogeneity. A significant Q_between (*p* > 0.05) indicates that the observed differences in effect sizes between subgroups are due to random variation, whereas a significant Q_between (*p* ≤ 0.05) suggests that subgroup membership may be part of the variability in the pooled estimates. Publication bias was assessed using Egger's regression test to evaluate funnel plot asymmetry, providing a quantitative measure of potential small-sample effects. In addition, funnel plots were visually inspected to identify visual deviations in symmetry. A *p* value < 0.05 in Egger's test was considered indicative of significant publication bias. The RStudio program (metaprop package), version 4.2, was used to analyze all data. Statistical significance was defined as *p* values < 0.05.

## Results

### Search results

**Fig. 1:**
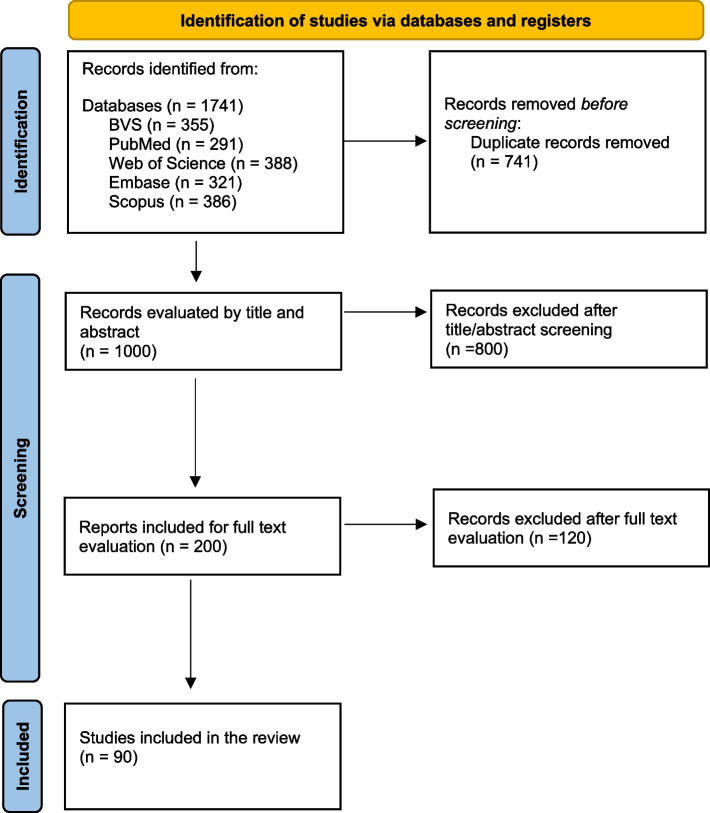
Flowchart of articles selected based on PRISMA 2020

The details of the selection process are presented in Fig. 1.

### Search characteristics

The 90 articles analyzed totaled 64,699 women assessed for postpartum depression during the COVID-19 pandemic in 31 countries (Supplementary Table 2). Individual samples from each study ranged from 50 to 5,134 women between 2 weeks and 1 year postpartum. Of the articles, 63 were cross-sectional studies, and 27 were cohort studies, with 16 prospective cohorts and 6 retrospective cohorts. The HDI ranged from 0.942 (Germany) to 0.544 (Pakistan), while maternal mortality ranged from 2 (Italy and Poland) to 342 (Kenya) and the GII was between 0.916 (Norway) and 0.506 (Kenya). Further details about the included articles can be found in Supplementary Table 2.

### Postpartum depression prevalence

**Fig. 2:**
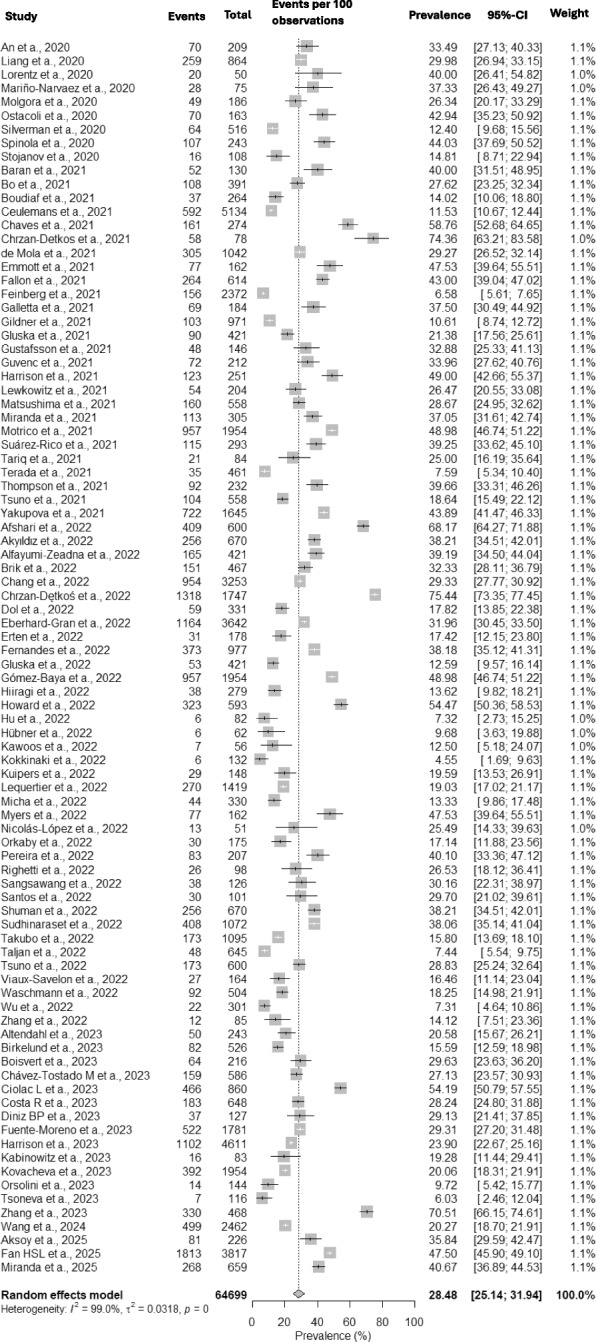
Forrest plot with overall prevalence of postpartum depression during the COVID-19 pandemic, with all 90 studies

Based on the meta-analysis of 90 articles, the global prevalence of postpartum depression during the COVID-19 pandemic was 28.48% [25.14—31.94; I^2^ = 99%], as can be seen in Fig. 2. The high heterogeneity among the included articles, which can be observed through the funnel plot in Suppl Fig. 1, can be explained by the great regional and sociocultural diversity of the countries where the studies were conducted, in addition to the different cutoff points of the instruments for PPD screening. The influence of these factors on the prevalence of PPD, especially during the pandemic period, will be further addressed in the discussion section. In the analysis of asymmetry detected by the Egger test, statistical significance was observed with *p*-value 0.9029. The main instrument used, present in 78 studies (86.66% of the total), was the Edinburgh Postnatal Depression Scale (EPDS). The EPDS diagnostic cut-off for PPD ranged from ≥ 8 to ≥ 14 points on the questionnaire. The ≥ 13 cutoff point was the most used for the diagnosis of PPD, present in 26 articles. The prevalence of PPD among studies with EPDS cutoff point ≥ 13 was 23.52% [18.96—28.40; I^2^ = 98.3%], (Suppl Fig. 2). In the analysis of asymmetry detected by the Egger test, statistical significance was observed with *p*-value 0.3448. Twelve articles used other screening/diagnostic scales for postpartum depression: CES-D [1], PHQ [6], DASS-21 [2], PDSS [3]; all scales are self-administered with responses in Likert scale format.

### Geographic analysis

**Fig. 3:**
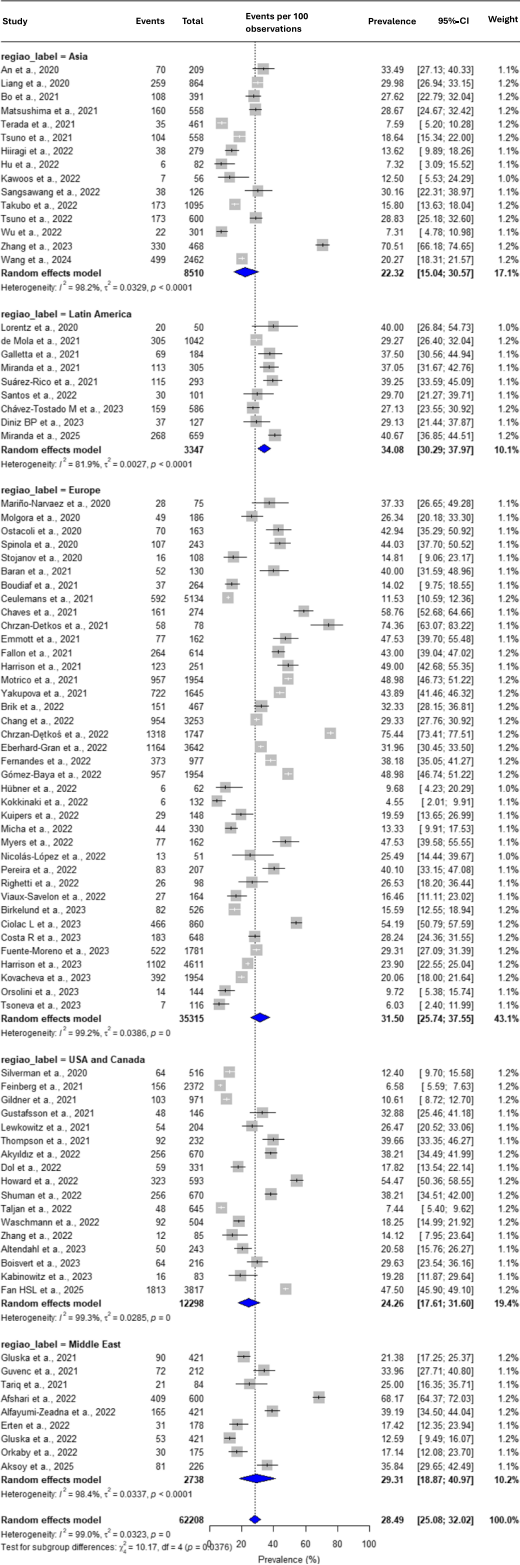
Forrest plot showing regional analysis of the prevalence of postpartum depression

To improve the data analysis, the 31 countries covered in the articles were grouped into 5 macro-regions according to the geographic proximity and continental division. Geographic variations in the prevalence of PPD were observed (Fig. 3) (Suppl Fig. 3), with the highest rate observed in Latin America [26, 45–52], with 34.08% [30.29—37.97; 9 articles; I2 = 81.9%], followed by Europe [25, 37, 53–86], with 31.50% [25.74—37.55; 38 articles; I2 = 99.2%], Middle East [27, 28, 87–93], with 29.31% [18.87—40.97; 9 articles; I2 = 98.4%], US/Canada [94–110], with 24.26% [17.61—31.60; 17 articles; I2 = 99.3%), Asia [111–124], with 22.32% [15.04—30.57; 15 articles; I2 = 98.2%]. There was only one study from the African continent [125], which analyzed the prevalence of PPD in Kenya (38.05%) and one study from Oceania [126], with an analysis of the prevalence in Australia (19.02%). In the analysis of asymmetry detected by the Egger test, statistical significance was observed with *p*-value 0.5897 (USA/Canada); 0.5066 (Europa); 0.9179 (Asia). There was a statistically significant difference when comparing the groups according to regional differences in PPD prevalence (*p*-value 0.0376).

### HDI

According to the HDI data from the UN Human Development Report (2021–2022) [42], the meta-analysis indicated a prevalence of 27.88% [24.21—31.70; I2 = 99.1%] for countries with a higher HDI (≥ 0.8) and a prevalence of 30.95% [23.15—39.33; I2 = 98.3%] for countries with a lower HDI (Suppl Fig. 4 and 5). In the analysis of asymmetry detected by the Egger test, statistical significance was observed with p-value 0.9326 (HDI ≥ 0.8); 0.7085 (HDI < 0.8). There was no statistically significant difference when comparing the groups (*p*-value 0.4922).

### COVID-19 case fatality rate

The postpartum depression rate was higher in countries with a higher COVID-19 case fatality rate: 32.56% [26.65—38.77; I2 = 99.2%] in countries where the CFR was greater than 1, versus 25.99% [22.16—30.00; I2 = 98.4%] in countries where the CFR was less than 1 (Suppl Fig. 6 and 7). There was no statistically significant difference when comparing the groups (*p*-value 0.0713).

### Maternal mortality

To analyze the influence of mortality on the prevalence of postpartum depression, countries were divided into three groups according to their maternal mortality rates in ascending order. Group 1 includes countries with the lowest maternal mortality (< 10 maternal deaths per 100,000 live births), followed by Group 2 with intermediate mortality (between 10 and 50 maternal deaths), and finally, Group 3, which includes countries with the highest maternal mortality rates (> 50 maternal deaths). The maternal mortality prevalences obtained by the meta-analysis were 28.10% [23.28- 33.19; I2 = 99%] in group 1, 28.65% [23.27—34.34; I2 = 99.1%] in group 2, and 30.43% [25.02—36.12; I2 = 81.8%] in group 3, as can be seen in Suppl Fig. 8 and 9. In the analysis of asymmetry detected by the Egger test, statistical significance was observed with *p*-value 0.7881 (group 1); 0.7692 (group 2). There was no statistically significant difference when comparing the groups (*p*-value 0.8045).

### GII (Gender Inequality Index)

**Fig. 4:**
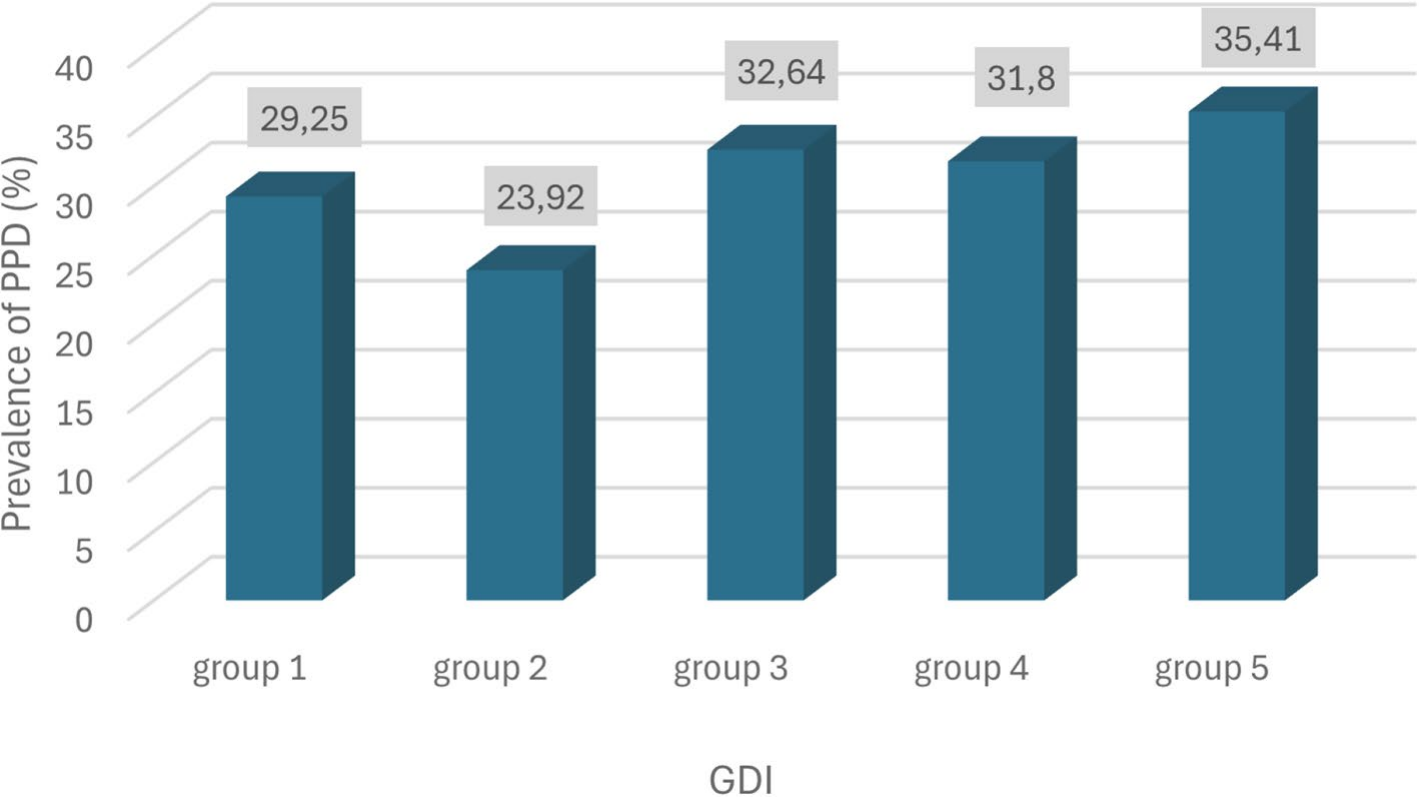
Graph showing the prevalence of postpartum depression according to the Gender Inequality Index

The meta-analysis was performed by dividing a sample of studies into five groups in increasing order of GII, that is, from countries with the lowest gender inequality (group 1) to countries with the highest inequality (group 5). The prevalence rates obtained were 29.25% [24.58—34.15; I^2^ = 99.1%] in group 1; 23.92% [17.64—30.82; I^2^ = 98.9%] in group 2; 32.64% [27.30—38.21; I^2^ = 87.7%] in group 3; 31.80% [27.77—35.98; I^2^ = 43%] in group 4 and, finally, 35.41% [14.06—60.33; I^2^ = 98.5%] in group 5. In the analysis of the asymmetry detected by the Egger test, statistical significance was observed with *p*-value 0.9702 (GII between 1–30) and 0.5224 (GII between 31–60). The graph showing the prevalence of postpartum depression according to the GII can be seen in Fig. 4, while the forrest and funnel plots can be found in Suppl Figs. 10 and 11. There was no statistically significant difference in the comparison of the groups (*p*-value 0.2906).

### Postpartum depression analysis by time

**Fig. 5:**
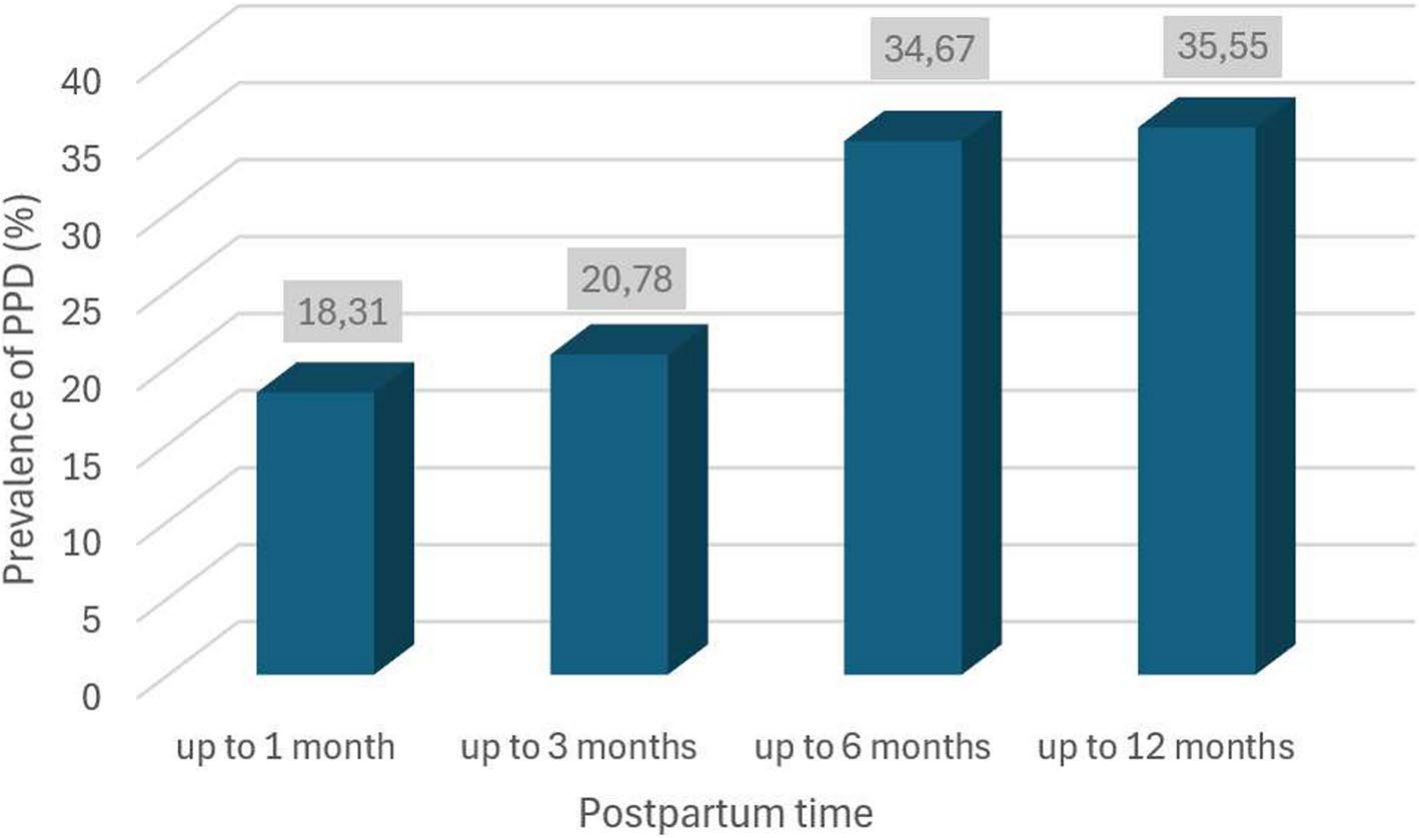
Graph with prevalence and standard deviation of postpartum depression according to time after birth

The prevalence rate of postpartum depression was also analyzed according to the time elapsed after delivery. To understand how PPD rates changed over time, four postpartum periods were evaluated: up to 1 month, up to 3 months, up to 6 months, and up to 12 months after delivery. There was evidence of a progressive increase in prevalence with postpartum time (Fig. 5), ranging from 18.31% [11.90—25.70, I2 = 90.9%] (up to 1 month), 20.78% [16.52—25.38, I2 = 97.8%] (up to 3 months), 34.67% [27.37—42.35, I2 = 98.8%] (up to 6 months) and 36.55% [29.72—43.66, I2 = 98.9%] (up to 12 months). In the analysis of the asymmetry detected by the Egger test, statistical significance was observed with *p*-value = 0.0361 (up to 3 months); 0.5312 (up to 6 months); 0.6841 (up to 12 months). Forrest and Funnel plots of the postpartum periods can be found in the Suppl Figs. 12 and 13. There was a statistically significant difference in the comparison between the groups (*p*-value < 0.0001).

### Risk factors and protective factors

Risk and protective factors were reported in 73 articles (54,051 women) and can be divided into two broad groups: factors directly related to COVID-19 and those unrelated. Factors directly related to COVID-19 encompass specific situations within this context. Those unrelated to the pandemic encompass both issues already established in the literature as influential in the development of PPD, as well as conditions with fewer citations, but which have also been shown to be important factors in women's mental health in the postpartum period.

Fifty-three different risk factors were identified, 16 of which were directly related to the COVID-19 pandemic, such as maternal COVID-19 infection and social isolation. Meanwhile, 37 risk factors were unrelated to the pandemic, such as a previous history of psychiatric illness and obstetric violence. Furthermore, 11 protective factors were identified, 2 of which were directly related to the pandemic and 9 were unrelated, such as the presence of social support, multiparity, among others. A table containing a detailed description of the risk and protective factors, number of citations and articles in which they were present can be found in Supplementary Table 3.

## Discussion

### Prevalence of postpartum depression before and during the pandemic

The overall prevalence of PPD during the COVID-19 pandemic found in this review was 28.48% [25.14—31.94; I^2^ = 99%; *p*-value 0.9029], significantly higher than rates reported before the pandemic. In pre-pandemic years, the prevalence of depressive symptoms in the perinatal period ranged between 6.5% and 25.8%, with variations depending on the region, postpartum period, and methodology used [113–116].

A meta-analysis of 291 studies from 56 countries, just before the pandemic [127], reported an average prevalence of 17.6% (95% CI: 16.6–18.8%). Another even larger meta-analysis, with 565 articles, described a very similar overall PPD rate of 17.22%; however, it included a few early pandemic articles [128]. Conversely, another meta-analysis, focusing only on previously healthy women and comprising 58 studies, established an average prevalence of 12% [129].

Compared with previous estimates, the prevalence observed here represents a 61% increase over the 17.6% reported in a large pre-pandemic meta-analysis [127] and a 137% increase over the lower estimate of 12% [128]. By contrast, an analysis of 22 studies [130] comparing PPD rates before and during the pandemic found an average increase of 81%, an intermediate result within this range.

Our study showed high heterogeneity (99%), which affects confidence in the pooled estimates, similar to other meta-analyses (73–99.3%). This is likely due to the inclusion of diverse diagnostic methodologies and various regional realities. The study with the lowest heterogeneity [131] only included articles that used the EPDS (Edinburgh Postnatal Depression Scale), a pattern also observed in pre-pandemic reviews [21, 128, 132].

To better understand the factors contributing to the increased rate of PPD during the COVID-19 pandemic, we decided to investigate risk factors, the influence of postpartum time on maternal illness, and conduct a more detailed study of regional differences. These aspects will be discussed in more detail below.

### Risk factors for postpartum depression

Our review investigated both risk and protective factors for PPD. Few systematic reviews have synthesized this dual perspective. Overall, the literature consistently points to three main clusters of risk factors: socioeconomic and demographic vulnerabilities, psychological and psychiatric history, and obstetric and medical conditions.

Before the pandemic, large-scale analyses showed that structural indicators such as income inequality, maternal and infant mortality, and long working hours for women were associated with higher PPD prevalence [127]. Other reviews reinforced the role of psychological/psychiatric antecedents—particularly personal or gestational depression—and psychosocial stressors as the most frequent predictors [21, 22, 133]. Some obstetric conditions, such as gestational diabetes, also emerged as relevant, although findings were heterogeneous [133].

During the pandemic, the literature emphasized a shift in emphasis toward contextual and social determinants. Systematic reviews consistently identified reduced social support, economic hardship, relationship strain, social isolation, and fear of infection as central risk factors [134–136]. The effect of restricted access to maternal and child health services was also repeatedly documented [135]. Importantly, these social restrictions were shown to amplify vulnerabilities across multiple domains—economic, familial, and emotional—converging to increase PPD risk.

Across periods, protective factors were remarkably consistent: the presence of robust social support networks (family, friends, or community), stable and supportive marital relationships, and adequate access to health and psychological care were associated with a reduction in PPD risk [135].

In our current study, we concur with several of these authors and identified 53 different risk factors for PPD, with the main ones being: prior history of psychiatric disorders such as depression and anxiety (cited in 13 articles); pandemic-related concerns (12 articles); maternal COVID-19 infection (11 articles); prematurity or low birth weight (7 articles); being a single woman without a partner (5 articles); social isolation (5 articles); lack of social support and low socioeconomic status (both cited in 4 articles). Additionally, 11 different protective factors were noted: effective social support (cited in 9 articles); high level of education (6 articles); continuity of obstetric care and breastfeeding practices (both in 4 articles).

Therefore, we can see that some risk factors observed during the pandemic were present before the COVID-19 outbreak, such as a personal history of depression and anxiety [137], being single and without partner or family support [138], as well as obstetric complications during pregnancy or childbirth [139]. Although these factors persisted, their impact may have increased during the pandemic. After all, it was a time of great stress, with a significant increase in anxiety [140, 141]. Furthermore, experiencing any medical complication during the pandemic was much more distressing, as medical assistance was focused on treating COVID-19 victims, with less attention given to other health problems. Moreover, requiring hospital admission during this stressful period was challenging, not only due to the difficulty in securing a hospital bed but also the fear of contracting SARS-CoV-2 during hospitalization. This situation led to delays in care for various types of patients [142]. It is no coincidence that interest in home births increased intentionally, leading to a higher risk of complications [143, 144].

The decrease in conceptions and births during the pandemic exemplifies the immense fear of becoming pregnant and giving birth during that time, especially when so little was known about the repercussions for both mother and child [145]. Despite a reduction in births, the number of available hospital beds decreased in many locations, creating a very real fear for numerous women [26]. Although not universally agreed upon for all scenarios, there appears to have been an increase in rates of low birth weight, prematurity, cesarean section, and hypertension in pregnancy, a fact that could be related to increased maternal stress or a change in the behavioral patterns of pregnant women [146].

Moreover, it is plausible that social isolation emerged as a novel risk factor, exacerbating previously recognized determinants such as lack of social support, domestic violence, and low socioeconomic status. Lockdowns and home confinement significantly restricted social interaction and contact with family and friends, thereby increasing loneliness and limiting access to social support. Confinement with a partner in an already strained relationship often created an environment of heightened insecurity and, in some cases, even domestic violence, which tended to worsen given the additional barriers to reporting such incidents during this period [147].

Undoubtedly, the pandemic worsened economic conditions, and this impact was greater the higher the socioeconomic vulnerability. Social distancing and lockdowns changed production and consumption patterns, with a consequent negative impact on businesses and gross domestic product, resulting in business closures and economic slowdown. This situation exposed enormous health inequalities in most countries due to the unequal distribution of wealth and/or resources. People from lower socioeconomic backgrounds had even less access to essential health services during this critical period, increasing not only financial insecurity but also health and healthcare insecurity [148]. This created an environment conducive to an increase in postpartum depression.

Following this reasoning, it is not surprising that the possibility of stable family relationships and effective social support emerged as protective factors, denoting a functional normality amidst the unfolding social catastrophe, thus configuring a very important aspect of resilience. Some advocate that family connection is the most crucial link for maintaining mental health, acting as a significant resilience factor [149].

Being able to continue obstetric care and successfully breastfeed and adequately care for the bond with the newborn would be two factors that go hand-in-hand in maintaining a functional normalcy. This could contribute to the mental balance of the postpartum woman, providing some degree of security amidst a new, unsettling, and frightening situation. Furthermore, there is extensive theorization regarding the importance of oxytocin secretion, which is involved in the physiology of lactation, for maternal mental health during the puerperium [150].

### Geographical distribution

Given the relevance of these risk and protective factors, it would be reasonable to assume a differentiated distribution of PPD rates based on the specific realities of each global region.

The 31 countries included in this study were divided into five macro-regions to better observe potential differences in PPD prevalence around the world. Two studies, one from Australia and one from Kenya, were analyzed individually because they were the sole representatives from their respective continents.

The highest rate was observed in Latin America (34.08%), while the lowest occurred in Asia (22.32%). The other regions were represented as follows, in descending order: Europe (31.50%), Middle East (29.31%), and USA/Canada (24.26%).

This distribution is somewhat different from that presented by a large meta-analysis [128], which included 565 articles published between 2000 and 2021, encompassing 80 countries and over 1.2 million women. The overall PPD rate in that meta-analysis was 17.22%, not significantly different from the articles described before 2010 (17.94%). The highest rates were found in Africa (average 23.13%, ranging from 13.62% in West Africa to 39.96% in Southern Africa), followed by South America (21.71%) and Central America (20%). Next came Asia (average 18.26%, ranging from 13.53% in Southeast Asia to 22.32% in South Asia). Finally, North America (17.01%) and Europe (average 14.81%, ranging from 12.91% in Western Europe to 16.62% in Eastern Europe).

In our meta-analysis, unfortunately, there was only one African study, from Kenya, showing one of the highest rates (38.05%). This prevents us from making an adequate comparison with the pre-pandemic reality. However, what is striking when comparing to that large 2021 meta-analysis [128] with ours is that the prevalence of postpartum depression in Asia was not significantly different before and during the pandemic. On the other hand, Europe, which had one of the lowest pre-pandemic PPD rates (14.8%), saw its rates surge to one of the highest during the pandemic (31.36%). Also notable is the large increase in prevalence in Latin America (from 21 to 34%), which went from second place in that large analysis [128] to first place in our data during the pandemic. Similarly, the significant increase in rates in North America (from 17 to 24%) is impressive. Such a shift in the behavior of these regions leads us to consider the epidemiology of COVID-19 itself, which saw its epicenter rapidly move from Asia, where it remained for a short time, to Central and Mediterranean Europe, and then to the Americas, where it persisted longer with a significant impact [151–155].

The only partial understanding of regional differences leads us to consider other explanatory possibilities that might underlie the division by continental region. Given that the environment of health, social, and economic insecurity could reinforce prior disparities and vulnerabilities, thereby increasing the risk of postpartum depression, we wanted to evaluate with the available data whether this correlation would exist in the studied populations. Our hypothesis, based on the opinion of some researchers [40, 156, 157], was that there would be higher rates of postpartum depression in areas with greater social inequality and inequity and greater economic vulnerability. To this end, we utilized sub-analysis through some of these indicators of socioeconomic inequality and vulnerability, as well as access to healthcare.

### HDI

One possible hypothesis to explain the difference in PPD prevalence among these regions would be their respective levels of socioeconomic development. In the present publication, this was assessed using the HDI, based on the UN Human Development Report (2021–2022) [42]. A decrease in PPD rates was observed with an increase in HDI, with rates of 27.88% for countries with a higher human development index (HDI ≥ 0.8) and 30.95% for countries with a lower index (HDI < 0.8), which could partially explain the difference between regions.

However, if the level of socioeconomic development alone determined PPD rates, we would expect the Middle East and Asia to have higher PPD rates than regions like Europe, the United States, and Canada. This was not observed. If there had been other African countries in the sample, a trend of high prevalence might have been perceived, but we only had one African article to consider. On the other hand, Latin America stood out with the highest rates. We could, therefore, consider that the countries included in the studies on Asia and the Middle East might not be representative of the full range of economic and social development in those regions, which are, in fact, quite unequal in this regard. Thus, the difference in rates may be explained by the heterogeneity of how the COVID-19 pandemic and its associated problems affected each region.

Other authors have conducted evaluations similar to ours. A review [158], examining 15 studies, observed a pooled prevalence of 30.5% among high-income countries such as the United States, Italy, Spain, and Japan, and 31.5% in low- and middle-income countries, including Mexico, China, Pakistan, and India. This classification, distinguishing between high and low income, was based on World Bank criteria, considering gross national income per capita—thus, focusing solely on the financial aspect. The observed difference, with lower prevalence in wealthier countries, aligns with our findings.

However, this same World Bank classification had been used previously, noting not only a lower overall prevalence but also a much greater difference between countries based on income. Another review [159], for instance, analyzed data from 96 publications and identified a prevalence of 13.1% in low/middle-income countries versus 11.4% in high-income countries. This calculated an Odds Ratio of 1.8 (1.4–2.2), indicating an almost twofold risk of perinatal depression in poorer countries. Thus, there appears to have been a smaller impact of per capita income on the difference in postpartum depression between countries during the pandemic.

We understand that the HDI provides more comprehensive information regarding disparities between countries. Countries with a lower HDI tend to exhibit higher rates of PPD, likely due to factors such as reduced access to health services and limited social support, in addition to greater socioeconomic challenges.

### Maternal mortality

Reflecting further on the significance of the HDI, we considered that the maternal mortality ratio could help us assess regional inequalities in healthcare access. After all, scientific literature indicates that inadequate access to health services is strongly associated with maternal mortality. Factors such as delays in seeking care, resource scarcity, low quality of services, and socioeconomic inequalities contribute significantly to this scenario [160]. Undoubtedly, exposure to life-threatening risks brings fear and a loss of control, impacting mental health.

Indeed, a previous meta-regression study [127] had already indicated a correlation between high maternal mortality rates and high postpartum depression rates in 56 countries studied. When combined with other parameters of social and gender inequity, mortality can explain 73% of the variation in PPD rates among countries.

This meta-analysis revealed that the prevalence of PPD is higher in countries with high maternal mortality. The PPD rate was 28.10% in countries with low maternal mortality (group 1), rose to 28.65% in those with prevalent mortality (group 2), and reached 30.43% in those with the highest rates (group 3). This suggests that the quality of maternal health care, which includes prenatal, labor, and postpartum care, may influence the prevalence of PPD. Furthermore, there was an increase in maternal morbidity and mortality during the pandemic [161], with a greater impact in regions with limited resources, highlighting preexisting disparities and barriers to access to health care even before the 2019 pandemic [162–164]. This worsening of health conditions, especially for women during childbirth and the postpartum period, may have become a relevant risk factor for the development of postpartum depression.

The increase in maternal mortality can also be explained by the documented reduction in the quality of prenatal care offered to pregnant women during the 2019 pandemic [165]. It is known that reduced access to prenatal care directly impacts the rates of unfavorable birth outcomes, such as maternal mortality.

### COVID-19 case fatality rate

If maternal mortality during this period couldn't explain the rates of postpartum depression, another possibility is that the risk of death from COVID-19, rather than death due to childbirth, might have been impacting the mental health of this population. Our reflection was that the underlying fear of COVID-19 and its high CFR could be a new risk factor for PPD, existing only within the pandemic context.

Therefore, it seemed reasonable to study the behavior of PPD in relation to other public health aspects. The fear stemming from the pandemic should presumably be greater in regions of the globe facing greater difficulty in combating the disease, with higher fatality rates, which could negatively impact the mental health of these populations.

Thus, it would be logical to think that the pandemic and the way various countries and regions approached the problem could have influenced the population's sense of insecurity and stress, potentially offering an additional explanation for the regional differences observed here. In this sense, it is worth noting that the prevalence of PPD was higher precisely in the countries where the pandemic had a higher fatality rate due to COVID-19, being 32.56% [26.65—38.77; I2 = 99.2%] in countries with CFR > 1 and 25.99% [22.16—30.00; I2 = 98.4%] in countries with CFR < 1. The fear of dying, observing how the healthcare system proved inoperable in the face of a new viral threat, may have contributed to increased symptoms of anxiety and depression in this situation.

Furthermore, the hypothesis was raised that changes in the epicenter of the pandemic could have influenced regional PPD rates. Despite remaining a long-time epicenter of the pandemic with high SARS-CoV-2 infection rates, the United States and Canada successfully maintained well-functioning healthcare systems with low fatality rates. This was aided by both the preparedness of their healthcare systems and the effectiveness of the social distancing measures implemented. Secondarily, their economies remained more secure, with faster recovery than in other places.

Europe, in turn, was the pandemic's epicenter for a long time, with a significant impact on ICU occupancy and public health as a whole. Generally, isolation measures were quite strict, compromising interpersonal relationships and the economy.

Asia, on the other hand, is a vast and heterogeneous region, encompassing diverse realities. Although the pandemic began in China, health measures were intense and rigorous, leading to a rapid reduction in cases. Other countries in the region, like Japan and New Zealand, also demonstrated exemplary responses to the pandemic.

The Middle East also presents diverse realities among its constituent countries. A Saudi study points to low isolation rates among young people (around 10%) and a significant influence of isolation and socioeconomic conditions on the emergence of depression in this group [166].

Conversely, Africa faced various adversities during its pandemic response, with low isolation rates, primarily due to precarious sanitation conditions, informal settlements, poverty, food insecurity, extended families, and fragile healthcare systems [167].

Furthermore, the way each culture interprets feelings of unhappiness in the postpartum period is quite diverse, which may explain, at least in part, the different rates of postpartum depression across various parts of the globe [168].

In this regard, an interesting longitudinal study [169] that analyzed 289 Chinese pregnant women helps us reflect on the impact of fear. This study identified that fear of childbirth is significantly associated with PPD. Fear was categorized into four dimensions: concerns about the baby's health, loss of control, pain, and hospital interventions. The perceived partner response moderated this association, suggesting that emotional support can mitigate the negative effects of childbirth fear on postpartum mental health. Another Finnish population-based study, conducted before the pandemic, noted the same association between fear of childbirth and PPD [168]. This association between fear and PPD remained present during the pandemic. A study conducted in Portugal evaluated 207 postpartum women and found that fear of COVID-19 was a predictor of depressive and anxiety symptoms during this period. The study concluded that postpartum women were significantly vulnerable to the psychological effects of the pandemic [170].

Extrapolating to the results of the current meta-analysis, we can consider that countries with higher COVID-19 fatality rates triggered greater fear responses in their obstetric population, leading to higher PPD rates. Additionally, in the context of the pandemic, partner support during hospital stays was often absent, further contributing to the fear experienced by birthing people.

### GII (Gender Inequality Index)

The association of postpartum depression during the pandemic with parameters of gender inequality is interesting, as this unfair gender disparity can be related to factors such as intimate partner violence, disrespect and abuse during childbirth, and unfavorable socioeconomic conditions. A Brazilian study revealed that the presence of intimate partner violence during pregnancy was a risk factor for PPD [171]. Similarly, experiences of disrespect and abuse during childbirth are often linked to gender inequalities and an increased prevalence of PPD [172]. During the pandemic, childbirth conditions were no longer ideal, and there's evidence that domestic violence increased during isolation [173].

Our meta-analysis's GII analysis revealed that regions with greater gender inequality had higher PPD rates (group 5 with 35.41%) compared to regions with lower gender inequality (group 1 with 29.25%). Analyzing this result alone might suggest that the greater the gender inequality, the higher the risk of developing PPD. However, this wasn't exactly what was observed in this study. This is because when analyzing regions with a medium gender inequality index (group 3 with 32.64%), the resulting PPD prevalence was higher than that found in group 2 (23.92%) and group 4 (31.80%).

Given this, it's clear that PPD is related to gender inequality, but only partially. We can infer that high GII values, indicating high gender inequality, might signify a well-recognized risk scenario for postpartum depression: low education, limited access to reproductive health, low wages, and high maternal mortality. However, as discussed earlier, Northern Hemisphere countries with high per capita income and adequate socioeconomic development also suffered considerably during the pandemic, experiencing a significant increase in PPD prevalence.

Therefore, while it was insightful to seek explanations for the differences in global PPD rates through cultural analysis via the GII—and this systematic review is the first to establish some degree of correlation between PPD prevalence and gender inequality—this explanation was not entirely satisfactory. This is because it was not possible to fully understand the reason for the higher prevalence of PPD in regions with less gender inequality when compared to regions with average GII values, as occurs in group 3. Thus, further studies are needed to address this aspect for a better understanding of gender inequality's influence on PPD.

### Postpartum period

The postpartum time interval is a crucial variable in screening for and diagnosing PPD. In this study, we observed a progressive increase in PPD prevalence from the first month (18.31%) to the twelfth month (36.55%) postpartum. This progressive rise in PPD rates over time differs from findings in other articles and highlights that the choice of time interval for studying postpartum depression rates significantly impacts the results.

For example, a meta-analysis of 27 articles up to December 2020 [133] also revealed variations in PPD prevalence according to the postpartum period analyzed, but with a different pattern than what we found in our study. Their results showed a rate of 13.9% during the first 4 weeks postpartum, 15.3% at 6 weeks, 12.9% at 8 weeks, 17.4% at 12 weeks, and 13.6% at 24 weeks.

Furthermore, a systematic review conducted in 2004 [132] showed an increase in the estimated PPD prevalence from the first to the third month postpartum, ranging from 9.7% to 12.9%. Subsequently, there was a decrease in the rate between the fourth and twelfth month postpartum, varying from 10.6% to 6.5%. Thus, the highest prevalence observed by that study was during the third month after childbirth.

It's important to note that the pandemic context may have influenced the findings of this study, suggesting that as the postpartum period progresses, there's an overlap of the analyzed risk factors, such as social isolation, COVID-19 concerns, and financial impact. This could explain why our results showed a progressive increase in postpartum depression rates over the postpartum period.

Furthermore, an important factor to consider is survivorship bias, specifically differential dropout. This is because follow-up dropouts were not analyzed in studies that proposed serial assessments. Therefore, it is understood that there is a certain limitation to be considered when understanding the results of the temporal evolution of PPD rates.

### Strengths and limitations

A key strength of this study is the large number of included articles (90 studies), as such a significant sample lends greater reliability to the obtained results. Furthermore, this study was the first to conduct a comprehensive evaluation of the mental health of postpartum women during the pandemic, correlating various factors. Beyond the geographical analysis, already present in other articles, a more detailed assessment was also performed regarding the impact of socioeconomic status, health indicators (COVID-19 fatality rate and maternal mortality), and cultural and political issues, through the analysis of gender inequality. Important findings with practical implications were obtained, which can guide public health policies and budgetary management in future health emergencies, aiming to reduce disparities between countries and act to prevent the growth of PPD prevalence, as observed in this study.

This study has limitations, including the presence of articles that assessed PPD prevalence using a screening instrument for this condition, making it impossible to conclude an effective diagnosis or differentiate it from a prior comorbidity. Additionally, the high heterogeneity of the included articles may alter the precision of the results, affecting the confidence in the pooled estimates.

The comparison of pre-pandemic meta-analyses is complicated by differences in sample sizes, inclusion criteria, outcome definitions, statistical transformations, and modelling approaches, all of which may influence the pooled estimates and confidence intervals and limit direct comparability across reviews. To address these variations and for between-study heterogeneity, a random-effects model was applied. The Freeman-Tukey double arsine transformation was used to stabilize variances and accommodate studies reporting very low or very high prevalence values with pooled estimates subsequently back-transformed to the original proportion scale for interpretation.

There were also few studies on PPD prevalence from countries in the Middle East, Africa, and Oceania, as well as in developing countries. The limited regional data influenced the interpretation of the results, and it is important to consider certain biases, such as selection bias and underreporting. This is because most of the included studies are from developed countries, which tend to have better healthcare systems, and a higher rate of diagnosis and treatment is expected, unlike developing countries, which may be associated with higher rates of underreporting.

### Implications for practice

The findings of this study indicate a higher prevalence of PPD in countries with lower HDI and higher rates of maternal mortality, COVID-19 fatality, and gender inequality. Furthermore, a progressive increase in PPD rates was observed over time in the postpartum period. The practical implications of these results, which can reduce the impact of public health emergencies like the pandemic on women's postpartum mental health, are highlighted below.

Moreover, gender inequality also manifests in socioeconomic disparities and a lack of social support, factors that increase the risk of PPD. One study demonstrated that partner support during pregnancy mediates social inequalities in PPD [174]. The lack of adequate support, which certainly worsened during the pandemic, can exacerbate the effects of gender inequalities on maternal mental health. Our study provides further evidence of the importance of continuous advocacy for women's rights, especially in conditions of significant social vulnerability and limited access to healthcare.

Therefore, especially in times of health crisis, adequate training for healthcare professionals in PPD diagnosis and treatment is necessary, along with the importance of identifying women at higher risk of developing this disorder during prenatal consultations. Online consultations or group therapies are strategies that could potentially be adopted for high-risk women. Increased media coverage on what postpartum depression is, its high prevalence, symptoms, and consequences, as well as the need to seek professional help if identified, would also be beneficial. Mental health support networks should be promoted, as the present study identified that the lack of social support is an important risk factor for the development of PPD.

Thus, it is understood that countries with higher maternal mortality, higher COVID-19 fatality rates, and lower HDI likely have weaker health systems, which could explain the higher PPD rate observed in these regions. It is therefore understood that there is a need for better redistribution of resources during periods of public health emergencies, such as pandemics and environmental disasters. Countries with distinct realities have distinct needs and distinct risks regarding mental health. At the onset of the pandemic, the WHO (World Health Organization) sought to play this central role in promoting global health, coordinating actions among countries and organizations, and drawing attention to a potential and greater risk of mental illness. They predicted that the pandemic would be a challenge to psychological resilience, especially for vulnerable populations [175]. However, not all countries heeded this warning and did not prepare adequately. In this post-pandemic period, where some leaders underestimate the WHO's leadership, it is even more crucial to emphasize the organization's leading role in this process, coordinating actions, and promoting equity in the distribution of resources, such as supplies, vaccines, protective equipment, among others. In other global health emergencies, this coordination will be essential.

A progressive increase in PPD rates was also observed as time passed after childbirth. We could hypothesize that in the context of the pandemic, the longer the postpartum period, the less social support a mother might receive, leaving her increasingly alone with household chores and newborn care. And the more time spent under these circumstances, coupled with the stress, fear, and isolation of the pandemic, the greater the mental distress.

Thinking this way, we could conjecture that efforts to monitor the mental health of postpartum women could be extended for a longer duration during public health emergencies, potentially increasing the length of maternity leave during pandemics, coupled with active outreach to these patients by the healthcare system. Another possible measure could be the implementation of public policies that encourage women to remain employed after returning from maternity leave. A Brazilian study [176] involving 247,455 women revealed that after 24 months postpartum, 48% of women left their jobs, mostly due to employer decisions. This impact of motherhood on women's professional lives interferes with the GII, as it is calculated based on parameters related to the labor market, reproductive health, and financial independence.

It is important to consider that while the suggested measures require financial investment, such actions are justified by the economic impact of the illness itself. PPD can lead to decreased productivity, absenteeism from work, and medical leave, negatively impacting family income and healthcare costs. The suggestions presented here aim to reduce the risk of developing PPD, especially during challenging times like the pandemic.

## Conclusion

The prevalence of postpartum depression during the COVID-19 pandemic was higher than the average rate reported by studies prior to this period, which may be a consequence of mothers' exposure to new risk factors identified as directly related to the pandemic. The main instrument used for the diagnosis of PPD was the Edinburgh Postnatal Depression Scale, and articles that used this tool showed a lower prevalence than studies that used other assessment resources. Furthermore, the rate was lower as the EPDS cutoff level increased. Additionally, a progressive increase in the PPD rate was identified according to the postpartum moment in which screening was performed, indicating a greater risk of developing this condition as this period increases.

The review pointed to the persistence of the influence of classic risk factors for PPD, such as lack of social support and a history of previous psychiatric illness, but also presented new factors directly related to the pandemic period, such as maternal COVID-19 infection and concern generated by the pandemic. Regarding socioeconomic differences, the review pointed out differences in the prevalence of PPD between the macro-regions considered, as well as the possible causes of heterogeneity, among which the socioeconomic development of each locality and the way this relates to the occurrence and response of each society to the pandemic stand out.

Pre-existing disparities in the quality of women's health care services were exacerbated by the pandemic context, with a higher prevalence of PPD in countries with lower HDI and higher COVID-19 case fatality rate, maternal mortality, and gender inequality. These indicators allowed for a more comprehensive analysis of this issue, allowing us to highlight that socioeconomic and cultural aspects have an influence on the development of this pathology. Furthermore, it made possible the discussion about implications for the practice of the results obtained, in order to survey possible ways to reduce the impact of health emergency situations on the mental health of women in the postpartum period, in addition to promoting improvements in maternal quality of life.

Additionally, other factors may have interfered with the measurement of PPD, such as the absence of sufficient studies that evaluate this pathology in regions of the Middle East and Africa, and sociocultural aspects that differently influence how women of different nationalities understand and respond to the EPDS questionnaire. Furthermore, a heterogeneous impact of the COVID-19 pandemic in different regions was noted.

This highlighted the need for future research conducted in developing countries, as well as in regions of Africa and Oceania. The importance of further studies exploring the socioeconomic and cultural aspects of postpartum depression is also recognized, as this meta-analysis was a pioneer in this regard. Thus, the results and discussion in this study provided concrete data to motivate specific policy actions to prevent mental illness among women in the postpartum period.

## Supplementary Information

Supplementary Material 1.

Supplementary Material 2.

Supplementary Material 3.

Supplementary Material 4.

Supplementary Material 5.

Supplementary Material 6.

Supplementary Material 7.

Supplementary Material 8.

Supplementary Material 9.

Supplementary Material 10.

Supplementary Material 11: Methodological Quality Assessment of the Included Reviews According to the JBI Critical Appraisal for Analytical Cross-Sectional Studies. [25, 27, 28, 37, 47, 48, 52, 55, 57, 62, 66, 69, 73, 78, 79, 84–89, 91–94, 96, 97, 101, 102, 104, 105, 108, 112–116, 118, 119, 122, 124–126, 130, 170]

Supplementary Material 12: Table with description of articles included in the meta-analysis. [25, 27, 28, 37, 47, 48, 52, 55, 57, 62, 66, 67, 69, 73, 75, 78–80, 84–94, 96, 97, 102–106, 108, 112–116, 118, 119, 122, 124–126, 130, 170]

Supplementary Material 13: Table with risk and protective factors for postpartum depression during the COVID-19 pandemic. [25, 27, 28, 37, 47, 48, 52, 55, 57, 62, 66, 69, 75, 79, 80, 84, 85, 87–89, 91–94, 96, 97, 102–104, 108, 112, 115, 116, 118, 119, 122, 124–126, 130, 170]

## Data Availability

No datasets were generated or analysed during the current study.
