# Supplementary figures and images for: Prevalence of postpartum depression in the COVID-19 pandemic and associated factors: systematic review and meta-analysis
Source: BMC Pregnancy Childbirth. 2026 Jan 20;26:157. doi: 10.1186/s12884-025-08262-z (PMC12903221; doi:10.1186/s12884-025-08262-z)

Standard Error

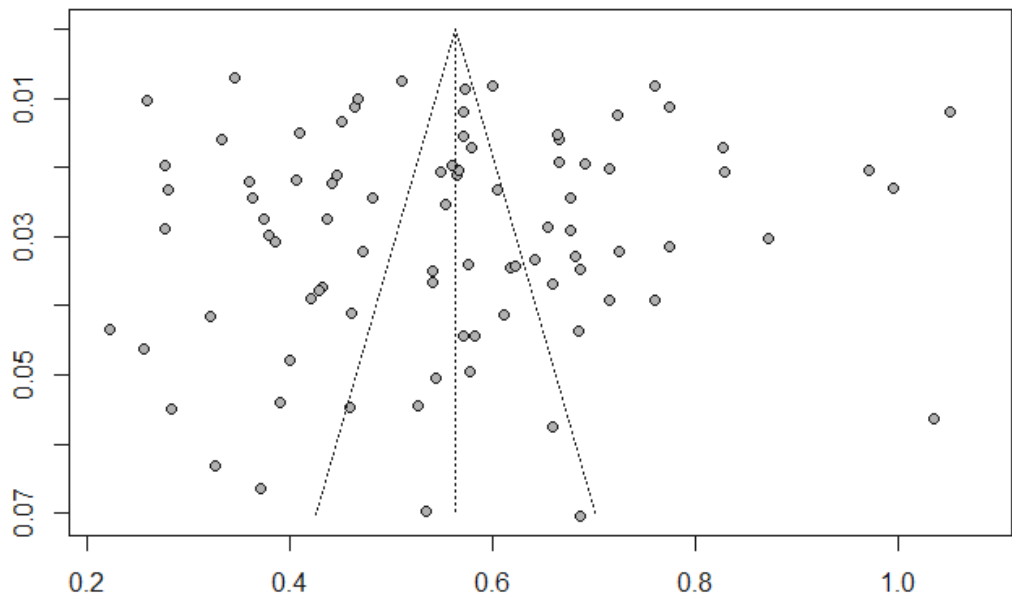

Freeman-Tukey Double Arcsine Transformed Proportion

Supplement: Supplementary file 1 — Supplementary Material 1. [file 12884_2025_8262_MOESM1_ESM.pdf]

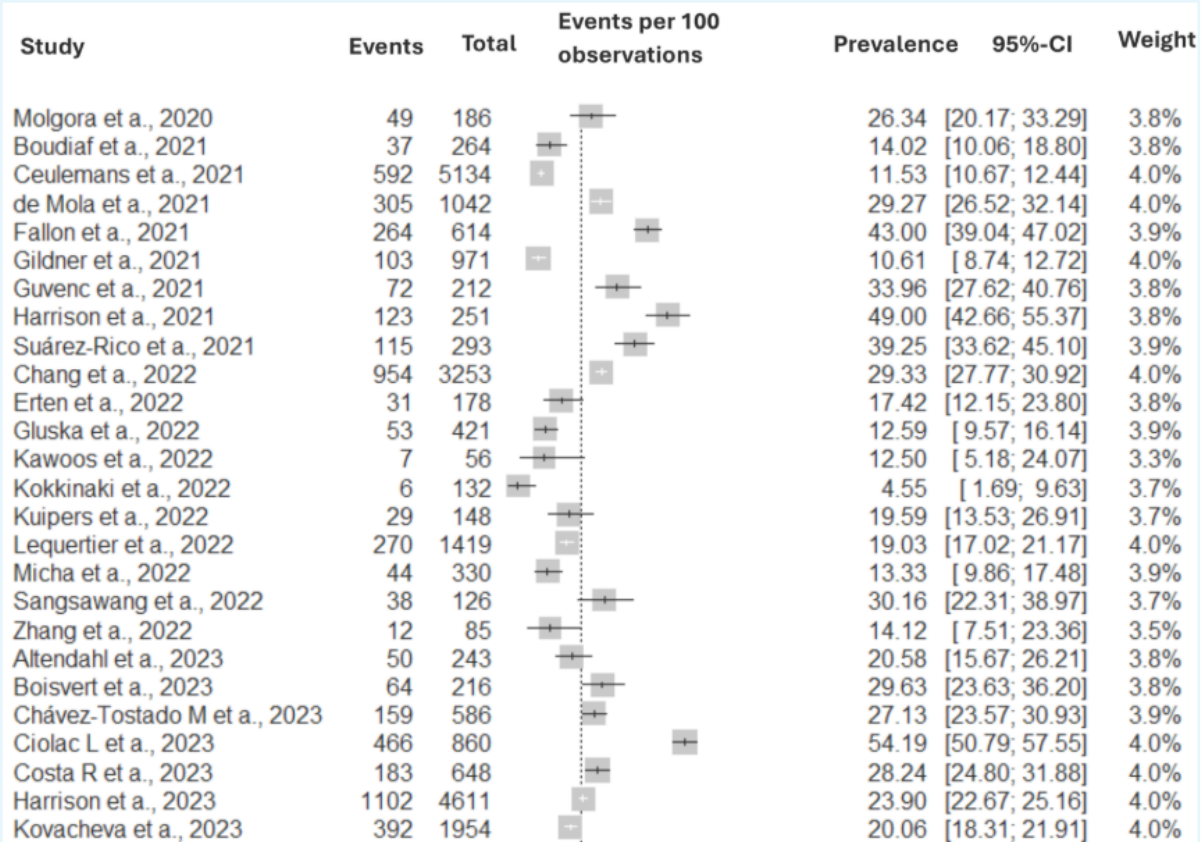

**Random effects model** **24233** **23.52 [18.96; 28.40] 100.0%**

Heterogeneity:  $I^2 = 98.3\%$ ,  $\tau^2 = 0.0199$ ,  $p < 0.0001$

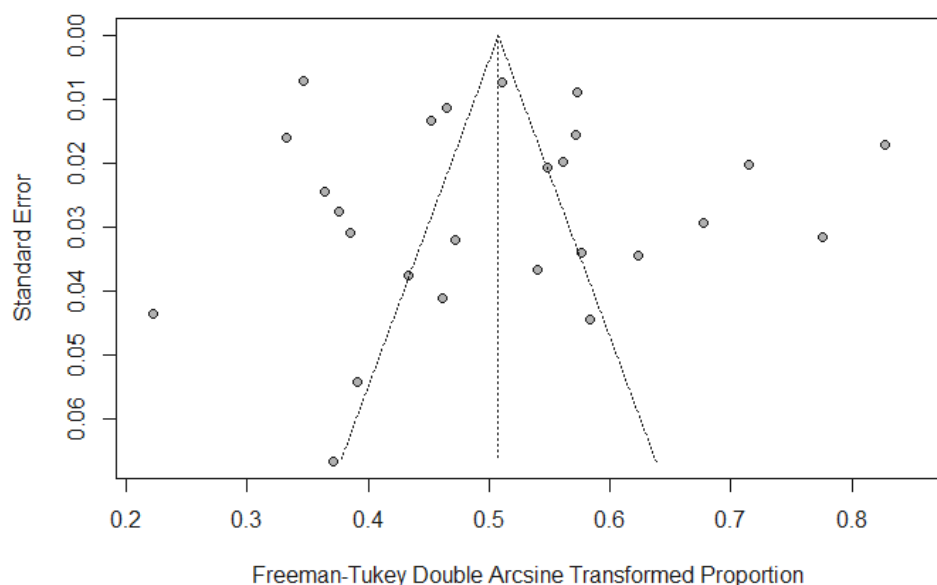

Supplement: Supplementary file 2 — Supplementary Material 2. [file 12884_2025_8262_MOESM2_ESM.pdf]

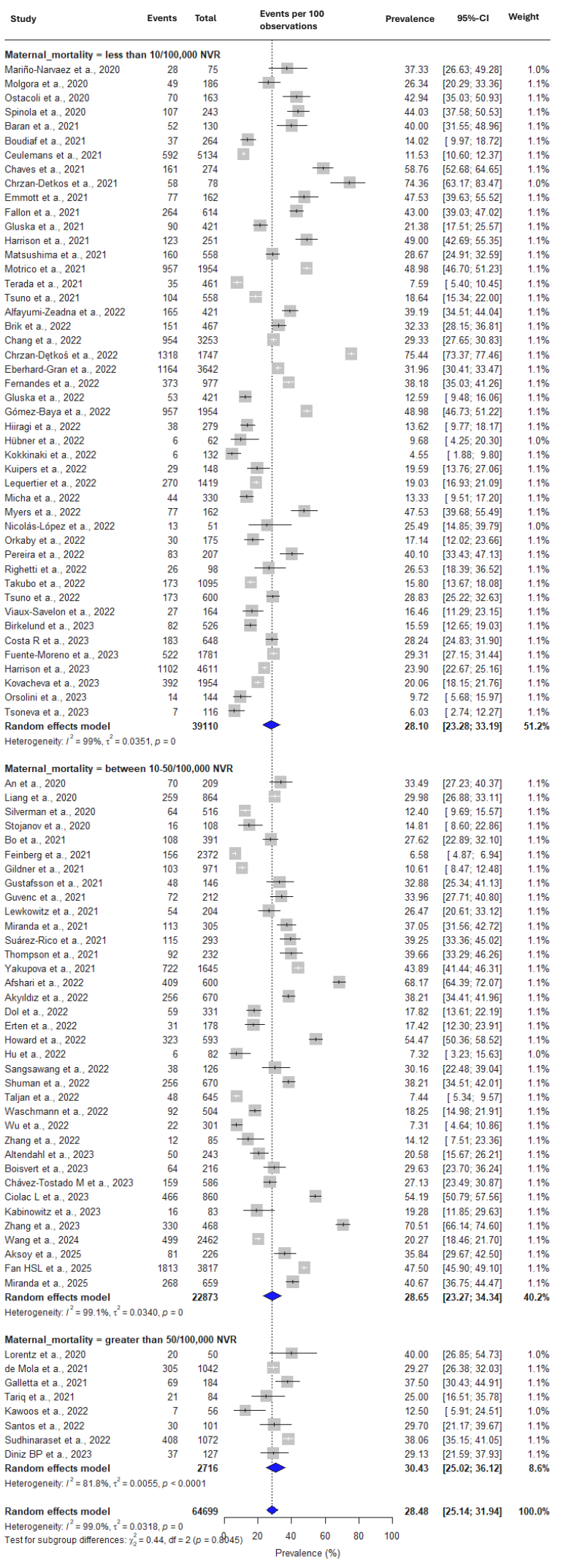

Supplement: Supplementary file 8 — Supplementary Material 8. [file 12884_2025_8262_MOESM8_ESM.pdf]
