# Supplementary material for: Prevalence of postpartum depression in the COVID-19 pandemic and associated factors: systematic review and meta-analysis
Source: BMC Pregnancy Childbirth. 2026 Jan 20;26:157. doi: 10.1186/s12884-025-08262-z (PMC12903221; doi:10.1186/s12884-025-08262-z)
Supplement: Supplementary file 3 — Supplementary Material 3. [file 12884_2025_8262_MOESM3_ESM.pdf]

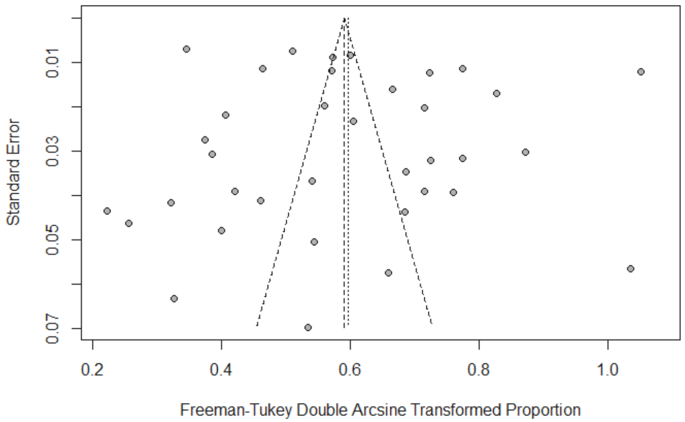

a)Funnel plot with prevalence of postpartum depression in Europe

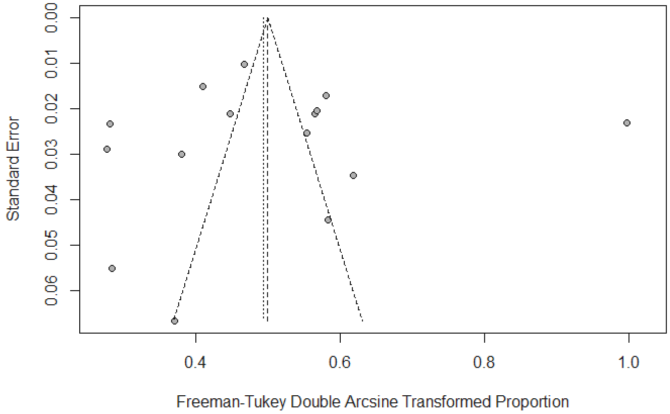

b)Funnel plot with prevalence of postpartum depression in Asia

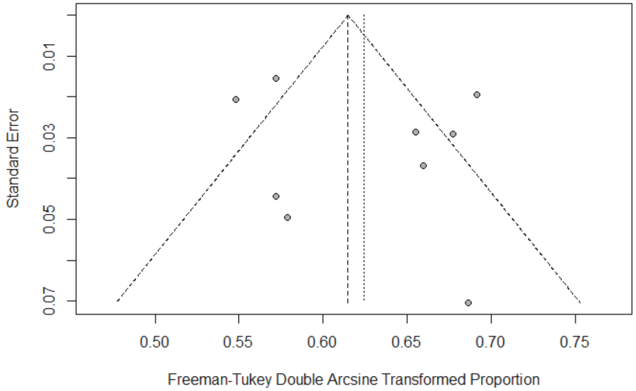

c)Funnel plot with prevalence of postpartum depression in Latin America

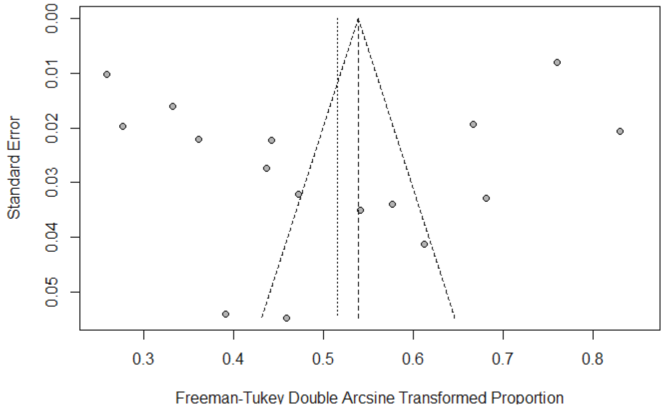

d)Funnel plot with prevalence of postpartum depression in USA and Canada

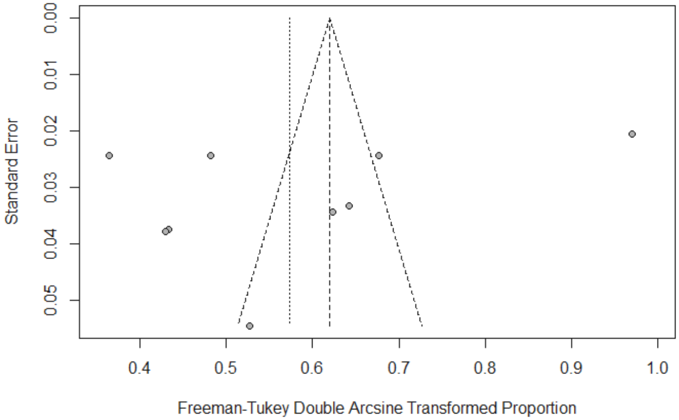

e)Funnel plot with prevalence of postpartum depression in Middle East
