# Supplementary material for: Prevalence of postpartum depression in the COVID-19 pandemic and associated factors: systematic review and meta-analysis
Source: BMC Pregnancy Childbirth. 2026 Jan 20;26:157. doi: 10.1186/s12884-025-08262-z (PMC12903221; doi:10.1186/s12884-025-08262-z)

Study	Events	Total	Prevalence	95%-CI	Weight
**HDI = < 0.800**					
An et a., 2020	70	209	33.49	[27.13; 40.33]	1.1%
Liang et a., 2020	259	864	29.98	[26.94; 33.15]	1.1%
Lorentz et a., 2020	20	50	40.00	[26.41; 54.82]	1.0%
Bo et a., 2021	108	391	27.62	[22.96; 32.15]	1.1%
de Mola et a., 2021	305	1042	29.27	[26.42; 32.06]	1.1%
Galletta et a., 2021	69	184	37.50	[30.47; 44.92]	1.1%
Suárez-Rico et a., 2021	115	293	39.25	[33.65; 45.10]	1.1%
Tariq et a., 2021	21	84	25.00	[16.45; 35.75]	1.1%
Afshari et a., 2022	409	600	68.17	[64.38; 72.04]	1.1%
Hu et a., 2022	6	82	7.32	[2.93; 15.40]	1.0%
Kawoos et a., 2022	7	56	12.50	[5.82; 24.46]	1.0%
Santos et a., 2022	30	101	29.70	[21.19; 39.68]	1.1%
Sudhinaraset et a., 2022	408	1072	38.06	[35.15; 41.05]	1.1%
Wu et a., 2022	22	301	7.31	[4.62; 10.84]	1.1%
Chávez-Tostado M et a., 2023	159	586	27.13	[23.28; 30.72]	1.1%
Diniz BP et a., 2023	37	127	29.13	[21.61; 37.94]	1.1%
Zhang et a., 2023	330	468	70.51	[66.22; 74.71]	1.1%
Wang et a., 2024	499	2462	20.27	[18.66; 21.88]	1.1%
**Random effects model**		**8972**	**30.95**	**[23.15; 39.33]**	**19.8%**
Heterogeneity: $I^2 = 98.3\%$, $\tau^2 = 0.0343$, $p < 0.0001$					
**HDI = ≥ 0.800**					
Mariño-Narvaez et a., 2020	28	75	37.33	[26.73; 49.28]	1.0%
Molgora et a., 2020	49	186	26.34	[20.17; 33.29]	1.1%
Ostacoli et a., 2020	70	163	42.94	[35.31; 50.92]	1.1%
Silverman et a., 2020	64	516	12.40	[9.67; 15.55]	1.1%
Spinola et a., 2020	107	243	44.03	[37.66; 50.52]	1.1%
Stojanov et a., 2020	16	108	14.81	[8.89; 23.06]	1.1%
Baran et a., 2021	52	130	40.00	[31.58; 48.96]	1.1%
Boudiaf et a., 2021	37	264	14.02	[10.02; 18.76]	1.1%
Ceulemans et a., 2021	592	5134	11.53	[10.61; 12.38]	1.1%
Chaves et a., 2021	161	274	58.76	[52.68; 64.64]	1.1%
Chrzan-Detkos et a., 2021	58	78	74.36	[63.05; 83.18]	1.0%
Emmott et a., 2021	77	162	47.53	[39.67; 55.50]	1.1%
Fallon et a., 2021	264	614	43.00	[38.93; 46.99]	1.1%
Feinberg et a., 2021	156	2372	6.58	[5.54; 7.58]	1.1%
Gildner et a., 2021	103	971	10.61	[8.61; 12.60]	1.1%
Gluska et a., 2021	90	421	21.38	[17.57; 25.62]	1.1%
Gustafsson et a., 2021	48	146	32.88	[25.49; 41.18]	1.1%
Guvenc et a., 2021	72	212	33.96	[27.68; 40.79]	1.1%
Harrison et a., 2021	123	251	49.00	[42.68; 55.35]	1.1%
Lewkowitz et a., 2021	54	204	26.47	[20.63; 33.13]	1.1%
Matsushima et a., 2021	160	558	28.67	[24.94; 32.62]	1.1%
Miranda et a., 2021	113	305	37.05	[31.67; 42.76]	1.1%
Motrico et a., 2021	957	1954	48.98	[46.74; 51.22]	1.1%
Terada et a., 2021	35	461	7.59	[5.31; 10.37]	1.1%
Thompson et a., 2021	92	232	39.66	[33.38; 46.28]	1.1%
Tsuno et a., 2021	104	558	18.64	[15.36; 22.02]	1.1%
Yakupova et a., 2021	722	1645	43.89	[41.47; 46.33]	1.1%
Akyıldız et a., 2022	256	670	38.21	[34.50; 42.00]	1.1%
Alfayumi-Zeadna et a., 2022	165	421	39.19	[34.53; 44.05]	1.1%
Brik et a., 2022	151	467	32.33	[28.08; 36.77]	1.1%
Chang et a., 2022	954	3253	29.33	[27.74; 30.90]	1.1%
Chrzan-Dętkoś et a., 2022	1318	1747	75.44	[73.62; 77.76]	1.1%
Dol et a., 2022	59	331	17.82	[13.39; 22.03]	1.1%
Eberhard-Gran et a., 2022	1164	3642	31.96	[30.11; 33.22]	1.1%
Erten et a., 2022	31	178	17.42	[12.08; 23.75]	1.1%
Fernandes et a., 2022	373	977	38.18	[35.04; 41.26]	1.1%
Gluska et a., 2022	53	421	12.59	[9.64; 16.20]	1.1%
Gómez-Baya et a., 2022	957	1954	48.98	[46.73; 51.22]	1.1%
Hiiragi et a., 2022	38	279	13.62	[9.72; 18.13]	1.1%
Howard et a., 2022	323	593	54.47	[50.37; 58.68]	1.1%
Hübner et a., 2022	6	62	9.68	[4.08; 20.19]	1.0%
Kokkinaki et a., 2022	6	132	4.55	[1.81; 9.74]	1.0%
Kuipers et a., 2022	29	148	19.59	[13.41; 26.84]	1.1%
Lequertier et a., 2022	270	1419	19.03	[16.78; 20.96]	1.1%
Micha et a., 2022	44	330	13.33	[9.59; 17.26]	1.1%
Myers et a., 2022	77	162	47.53	[39.69; 55.49]	1.1%
Nicolás-López et a., 2022	13	51	25.49	[14.97; 39.82]	1.0%
Orkaby et a., 2022	30	175	17.14	[12.06; 23.68]	1.1%
Pereira et a., 2022	83	207	40.10	[33.42; 47.13]	1.1%
Righetti et a., 2022	26	98	26.53	[18.39; 36.52]	1.1%
Sangsawang et a., 2022	38	126	30.16	[22.36; 38.99]	1.1%
Shuman et a., 2022	256	670	38.21	[34.51; 42.00]	1.1%
Takubo et a., 2022	173	1095	15.80	[13.60; 18.02]	1.1%
Taljan et a., 2022	48	645	7.44	[5.10; 9.34]	1.1%
Tsuno et a., 2022	173	600	28.83	[25.18; 32.60]	1.1%
Viaux-Savelon et a., 2022	27	164	16.46	[11.30; 23.15]	1.1%
Waschmann et a., 2022	92	504	18.25	[14.88; 21.83]	1.1%
Zhang et a., 2022	12	85	14.12	[7.93; 23.62]	1.1%
Altendahl et a., 2023	50	243	20.58	[15.78; 26.28]	1.1%
Birkelund et a., 2023	82	526	15.59	[12.61; 18.99]	1.1%
Boisvert et a., 2023	64	216	29.63	[23.54; 36.16]	1.1%
Ciolac L et a., 2023	466	860	54.19	[50.79; 57.55]	1.1%
Costa R et a., 2023	183	648	28.24	[24.84; 31.90]	1.1%
Fuente-Moreno et a., 2023	522	1781	29.31	[26.94; 31.27]	1.1%
Harrison et a., 2023	1102	4611	23.90	[22.67; 25.15]	1.1%
Kabinowitz et a., 2023	16	83	19.28	[11.63; 29.51]	1.1%
Kovacheva et a., 2023	392	1954	20.06	[18.05; 21.68]	1.1%
Orsolini et a., 2023	14	144	9.72	[5.63; 15.94]	1.1%
Tsoneva et a., 2023	7	116	6.03	[2.83; 12.34]	1.1%
Aksoy et a., 2025	81	226	35.84	[29.59; 42.47]	1.1%
Fan HSL et a., 2025	1813	3817	47.50	[45.90; 49.10]	1.1%
Miranda et a., 2025	268	659	40.67	[36.89; 44.53]	1.1%
**Random effects model**		**55727**	**27.88**	**[24.21; 31.70]**	**80.2%**
Heterogeneity: $I^2 = 99.1\%$, $\tau^2 = 0.0315$, $p = 0$					
**Random effects model**		**64699**	**28.48**	**[25.14; 31.94]**	**100.0%**
Heterogeneity: $I^2 = 99.0\%$, $\tau^2 = 0.0318$, $p = 0$					
Test for subgroup differences: $\chi^2_1 = 0.47$, df = 1 ($p = 0.4922$)					

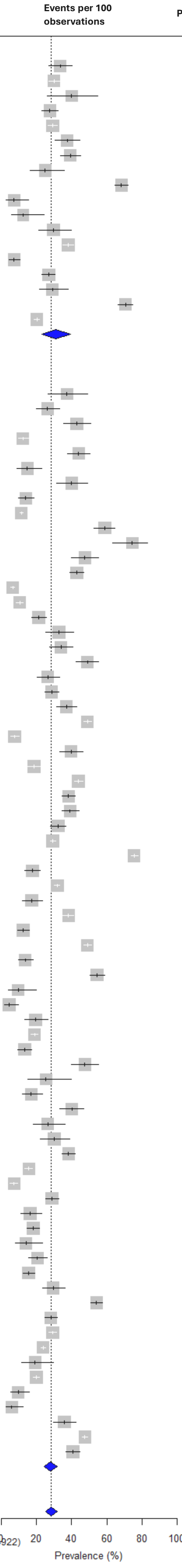

Supplement: Supplementary file 4 — Supplementary Material 4. [file 12884_2025_8262_MOESM4_ESM.pdf]
