# Supplementary material for: Prevalence of postpartum depression in the COVID-19 pandemic and associated factors: systematic review and meta-analysis
Source: BMC Pregnancy Childbirth. 2026 Jan 20;26:157. doi: 10.1186/s12884-025-08262-z (PMC12903221; doi:10.1186/s12884-025-08262-z)
Supplement: Supplementary file 5 — Supplementary Material 5. [file 12884_2025_8262_MOESM5_ESM.pdf]

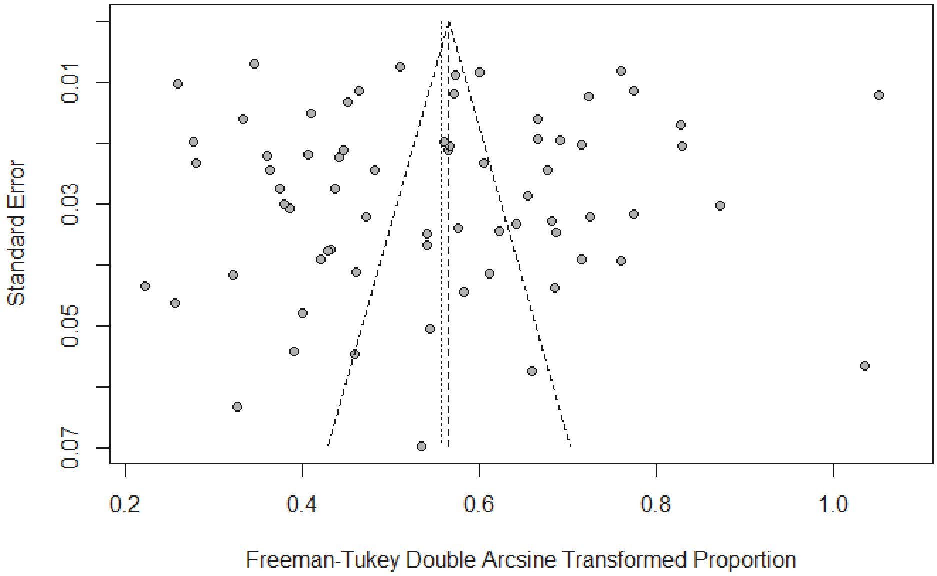

a) Funnel plot with prevalence of postpartum depression according to HDI in countries with HDI greater than or equal to 0.8.

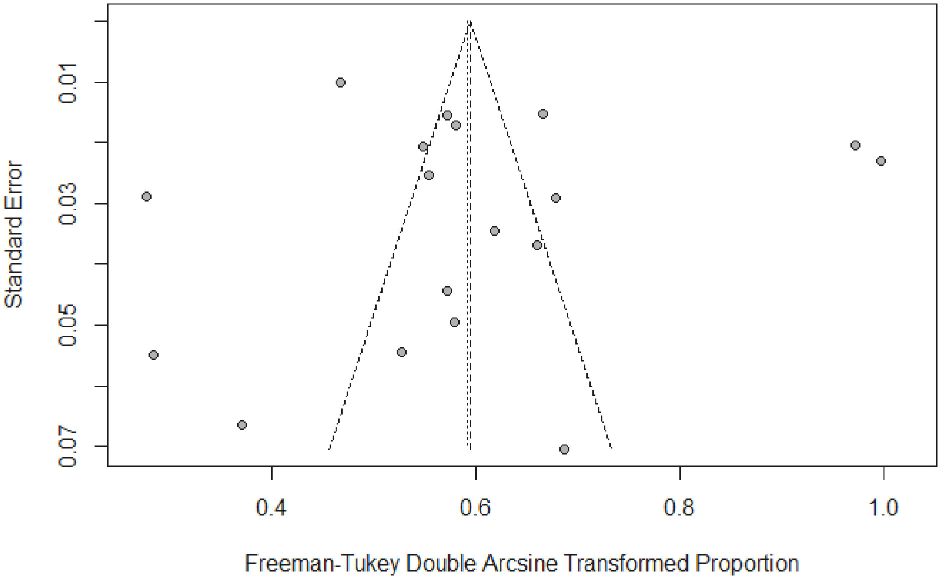

b) Funnel plot with prevalence of postpartum depression according to HDI in countries with HDI less than 0.8.
