# Supplementary material for: Prevalence of postpartum depression in the COVID-19 pandemic and associated factors: systematic review and meta-analysis
Source: BMC Pregnancy Childbirth. 2026 Jan 20;26:157. doi: 10.1186/s12884-025-08262-z (PMC12903221; doi:10.1186/s12884-025-08262-z)
Supplement: Supplementary file 6 — Supplementary Material 6. [file 12884_2025_8262_MOESM6_ESM.pdf]

Study	Events	Total	Events per 100 observations	Prevalence	95%-CI	Weight
**Lethality = < 1**						
An et a., 2020	70	209		33.49	[27.13; 40.33]	1.1%
Liang et a., 2020	259	864		29.98	[26.94; 33.15]	1.1%
Mariño-Narvaez et a., 2020	28	75		37.33	[26.28; 49.26]	1.0%
Molgora et a., 2020	49	186		26.34	[19.94; 33.16]	1.1%
Ostacoli et a., 2020	70	163		42.94	[35.24; 50.92]	1.1%
Spinola et a., 2020	107	243		44.03	[37.67; 50.52]	1.1%
Stojanov et a., 2020	16	108		14.81	[9.01; 23.14]	1.1%
Bo et a., 2021	108	391		27.62	[23.20; 32.31]	1.1%
Boudiaf et a., 2021	37	264		14.02	[9.85; 18.63]	1.1%
Ceulemans et a., 2021	592	5134		11.53	[10.38; 12.16]	1.1%
Chaves et a., 2021	161	274		58.76	[52.68; 64.63]	1.1%
Emmott et a., 2021	77	162		47.53	[39.67; 55.50]	1.1%
Fallon et a., 2021	264	614		43.00	[39.06; 47.02]	1.1%
Gluska et a., 2021	90	421		21.38	[17.51; 25.58]	1.1%
Guvenc et a., 2021	72	212		33.96	[27.44; 40.69]	1.1%
Harrison et a., 2021	123	251		49.00	[42.69; 55.35]	1.1%
Matsushima et a., 2021	160	558		28.67	[24.84; 32.55]	1.1%
Motrico et a., 2021	957	1954		48.98	[46.73; 51.22]	1.1%
Terada et a., 2021	35	461		7.59	[5.42; 10.47]	1.1%
Tsuno et a., 2021	104	558		18.64	[15.37; 22.02]	1.1%
Alfayumi-Zeadna et a., 2022	165	421		39.19	[34.52; 44.05]	1.1%
Brik et a., 2022	151	467		32.33	[28.10; 36.78]	1.1%
Chang et a., 2022	954	3253		29.33	[27.62; 30.80]	1.1%
Eberhard-Gran et a., 2022	1164	3642		31.96	[30.36; 33.43]	1.1%
Erten et a., 2022	31	178		17.42	[12.21; 23.84]	1.1%
Fernandes et a., 2022	373	977		38.18	[35.06; 41.27]	1.1%
Gluska et a., 2022	53	421		12.59	[9.60; 16.16]	1.1%
Gómez-Baya et a., 2022	957	1954		48.98	[46.73; 51.22]	1.1%
Hiiragi et a., 2022	38	279		13.62	[9.94; 18.31]	1.1%
Hu et a., 2022	6	82		7.32	[3.12; 15.55]	1.0%
Hübner et a., 2022	6	62		9.68	[3.81; 20.01]	1.0%
Kokkinaki et a., 2022	6	132		4.55	[1.93; 9.84]	1.1%
Kuipers et a., 2022	29	148		19.59	[13.62; 26.97]	1.1%
Lequertier et a., 2022	270	1419		19.03	[16.98; 21.14]	1.1%
Micha et a., 2022	44	330		13.33	[9.95; 17.56]	1.1%
Myers et a., 2022	77	162		47.53	[39.69; 55.49]	1.1%
Nicolás-López et a., 2022	13	51		25.49	[14.95; 39.82]	1.0%
Orkaby et a., 2022	30	175		17.14	[12.00; 23.65]	1.1%
Pereira et a., 2022	83	207		40.10	[33.41; 47.12]	1.1%
Righetti et a., 2022	26	98		26.53	[18.43; 36.54]	1.1%
Sangsawang et a., 2022	38	126		30.16	[22.51; 39.05]	1.1%
Takubo et a., 2022	173	1095		15.80	[13.61; 18.03]	1.1%
Tsuno et a., 2022	173	600		28.83	[25.27; 32.66]	1.1%
Viaux-Savelon et a., 2022	27	164		16.46	[11.16; 23.05]	1.1%
Wu et a., 2022	22	301		7.31	[4.74; 10.95]	1.1%
Birkelund et a., 2023	82	526		15.59	[12.58; 18.96]	1.1%
Costa R et a., 2023	183	648		28.24	[24.83; 31.90]	1.1%
Fuente-Moreno et a., 2023	522	1781		29.31	[27.13; 31.43]	1.1%
Harrison et a., 2023	1102	4611		23.90	[22.63; 25.12]	1.1%
Kovacheva et a., 2023	392	1954		20.06	[17.94; 21.58]	1.1%
Orsolini et a., 2023	14	144		9.72	[5.03; 15.47]	1.1%
Tsoneva et a., 2023	7	116		6.03	[2.08; 11.70]	1.1%
Zhang et a., 2023	330	468		70.51	[66.24; 74.74]	1.1%
Wang et a., 2024	499	2462		20.27	[18.50; 21.73]	1.1%
Aksoy et a., 2025	81	226		35.84	[29.67; 42.50]	1.1%
**Random effects model**		**42782**		**25.99**	**[22.16; 30.00]**	**61.2%**
Heterogeneity: I^2 = 98.4%, τ^2 = 0.0273, p = 0						
**Lethality = > 1**						
Lorentz et a., 2020	20	50		40.00	[26.79; 54.74]	1.0%
Silverman et a., 2020	64	516		12.40	[9.52; 15.42]	1.1%
Baran et a., 2021	52	130		40.00	[31.30; 48.94]	1.1%
Chrzan-Detkos et a., 2021	58	78		74.36	[63.12; 83.35]	1.0%
de Mola et a., 2021	305	1042		29.27	[26.43; 32.07]	1.1%
Feinberg et a., 2021	156	2372		6.58	[5.19; 7.24]	1.1%
Galletta et a., 2021	69	184		37.50	[30.44; 44.91]	1.1%
Gildner et a., 2021	103	971		10.61	[8.38; 12.39]	1.1%
Gustafsson et a., 2021	48	146		32.88	[25.47; 41.18]	1.1%
Lewkowitz et a., 2021	54	204		26.47	[20.67; 33.15]	1.1%
Miranda et a., 2021	113	305		37.05	[31.66; 42.76]	1.1%
Suárez-Rico et a., 2021	115	293		39.25	[33.65; 45.11]	1.1%
Tariq et a., 2021	21	84		25.00	[16.53; 35.79]	1.1%
Thompson et a., 2021	92	232		39.66	[33.28; 46.26]	1.1%
Yakupova et a., 2021	722	1645		43.89	[41.46; 46.32]	1.1%
Afshari et a., 2022	409	600		68.17	[64.30; 71.92]	1.1%
Akyıldız et a., 2022	256	670		38.21	[34.36; 41.93]	1.1%
Chrzan-Dętkoś et a., 2022	1318	1747		75.44	[73.44; 77.54]	1.1%
Dol et a., 2022	59	331		17.82	[13.89; 22.41]	1.1%
Howard et a., 2022	323	593		54.47	[50.36; 58.56]	1.1%
Kawoos et a., 2022	7	56		12.50	[5.86; 24.48]	1.0%
Santos et a., 2022	30	101		29.70	[21.27; 39.70]	1.1%
Shuman et a., 2022	256	670		38.21	[34.51; 42.01]	1.1%
Sudhinaraset et a., 2022	408	1072		38.06	[35.04; 40.98]	1.1%
Taljan et a., 2022	48	645		7.44	[5.58; 9.79]	1.1%
Waschmann et a., 2022	92	504		18.25	[15.04; 21.96]	1.1%
Zhang et a., 2022	12	85		14.12	[7.50; 23.36]	1.1%
Altendahl et a., 2023	50	243		20.58	[15.80; 26.30]	1.1%
Boisvert et a., 2023	64	216		29.63	[23.57; 36.17]	1.1%
Chávez-Tostado M et a., 2023	159	586		27.13	[23.39; 30.80]	1.1%
Ciolac L et a., 2023	466	860		54.19	[50.79; 57.56]	1.1%
Diniz BP et a., 2023	37	127		29.13	[21.62; 37.94]	1.1%
Kabinowitz et a., 2023	16	83		19.28	[11.72; 29.56]	1.1%
Fan HSL et a., 2025	1813	3817		47.50	[45.90; 49.10]	1.1%
Miranda et a., 2025	268	659		40.67	[36.89; 44.53]	1.1%
**Random effects model**		**21917**		**32.56**	**[26.65; 38.77]**	**38.8%**
Heterogeneity: I^2 = 99.2%, τ^2 = 0.0367, p = 0						
**Random effects model**		**64699**		**28.48**	**[25.14; 31.94]**	**100.0%**

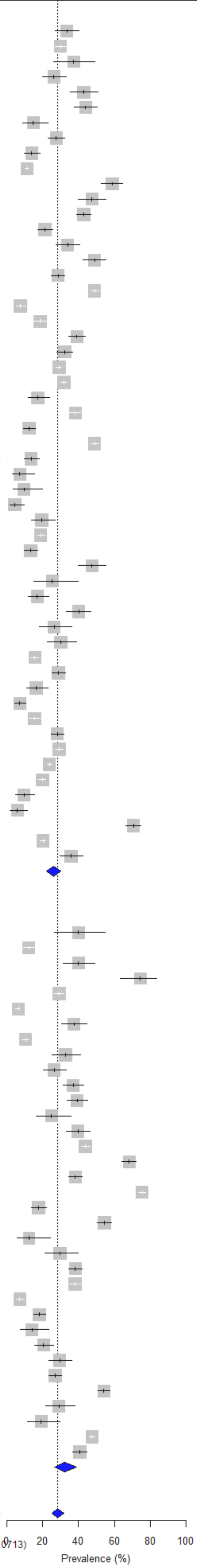

Heterogeneity: I^2 = 99.0%, τ^2 = 0.0318, p = 0
Test for subgroup differences: χ^2_1 = 3.25, df = 1 (p = 0.0713)
