# Supplementary material for: Prevalence of postpartum depression in the COVID-19 pandemic and associated factors: systematic review and meta-analysis
Source: BMC Pregnancy Childbirth. 2026 Jan 20;26:157. doi: 10.1186/s12884-025-08262-z (PMC12903221; doi:10.1186/s12884-025-08262-z)
Supplement: Supplementary file 7 — Supplementary Material 7. [file 12884_2025_8262_MOESM7_ESM.pdf]

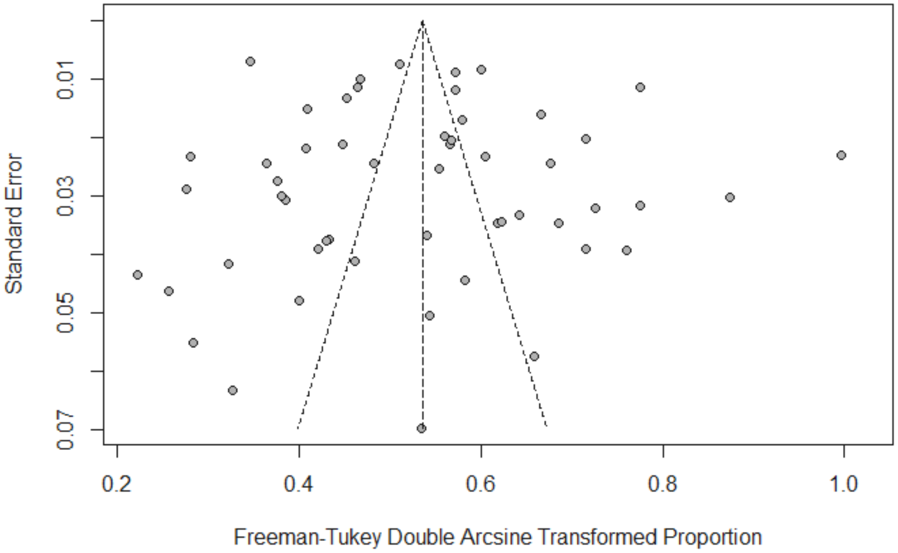

a)Funnel plot with prevalence of postpartum depression according to the fatality rate due to COVID-19 infection in countries with a fatality rate due to COVID-19 greater than 1

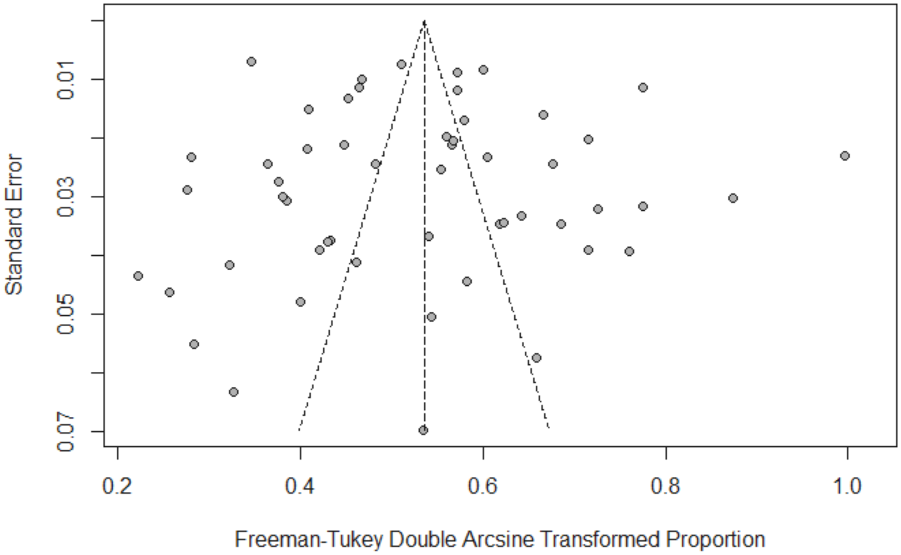

b)Funnel plot with prevalence of postpartum depression according to the fatality rate due to COVID-19 infection in countries with a fatality rate due to COVID-19 less than 1
