# Supplementary material for: Prevalence of postpartum depression in the COVID-19 pandemic and associated factors: systematic review and meta-analysis
Source: BMC Pregnancy Childbirth. 2026 Jan 20;26:157. doi: 10.1186/s12884-025-08262-z (PMC12903221; doi:10.1186/s12884-025-08262-z)
Supplement: Supplementary file 9 — Supplementary Material 9. [file 12884_2025_8262_MOESM9_ESM.pdf]

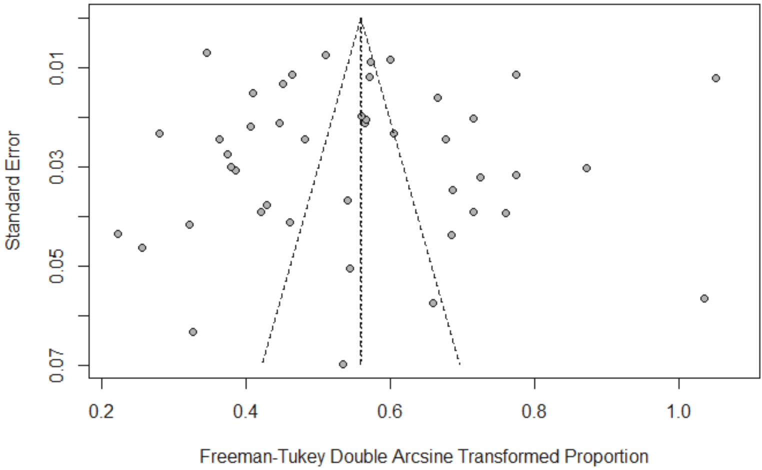

a)Funnel plot with prevalence of postpartum depression according to the maternal mortality rate of group 1 (less than 10/100,000 NVR)

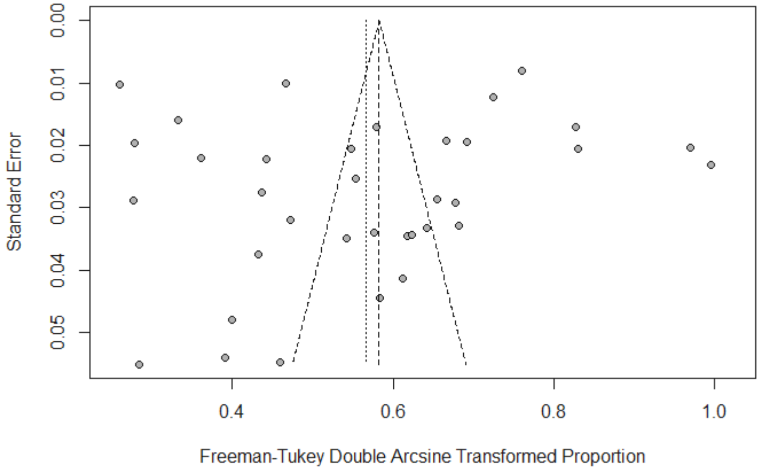

b)Funnel plot with prevalence of postpartum depression according to the maternal mortality rate of group 2 (between 10-50/100,000 NVR)

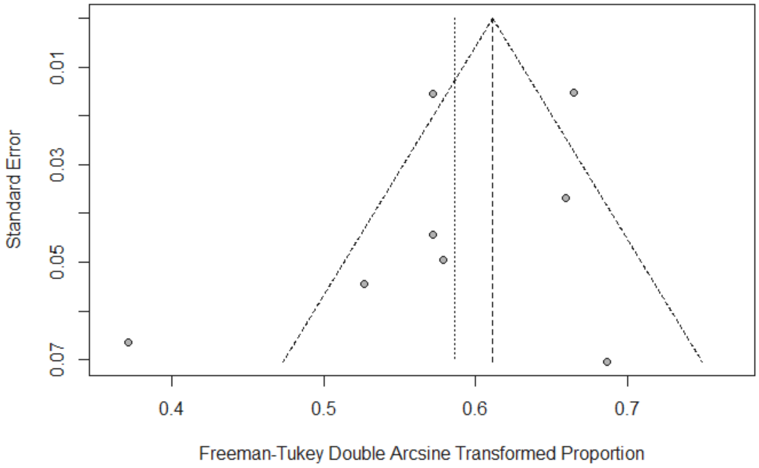

c)Funnel plot with prevalence of postpartum depression according to the maternal mortality rate of group 3 (greater than 50/100,000 NVR)
