# Supplementary material for: Prevalence of postpartum depression in the COVID-19 pandemic and associated factors: systematic review and meta-analysis
Source: BMC Pregnancy Childbirth. 2026 Jan 20;26:157. doi: 10.1186/s12884-025-08262-z (PMC12903221; doi:10.1186/s12884-025-08262-z)

| Study | Events | Total | Prevalence | 95%-CI | Weight |
|---|---|---|---|---|---|
| **GDI – GROUP 1** | | | | | |
| Mariño-Narvaez et a., 2020 | 28 | 75 | 37.33 | [26.63; 49.28] | 1.0% |
| Molgora et a., 2020 | 49 | 186 | 26.34 | [20.29; 33.36] | 1.1% |
| Ostacoli et a., 2020 | 70 | 163 | 42.94 | [35.03; 50.93] | 1.1% |
| Spinola et a., 2020 | 107 | 243 | 44.03 | [37.58; 50.53] | 1.1% |
| Boudiaf et a., 2021 | 37 | 264 | 14.02 | [10.00; 18.75] | 1.1% |
| Ceulemans et a., 2021 | 592 | 5134 | 11.53 | [10.44; 12.21] | 1.1% |
| Chaves et a., 2021 | 161 | 274 | 58.76 | [52.67; 64.62] | 1.1% |
| Chrzan-Detkos et a., 2021 | 58 | 78 | 74.36 | [63.10; 83.30] | 1.0% |
| Emmott et a., 2021 | 77 | 162 | 47.53 | [39.61; 55.53] | 1.1% |
| Fallon et a., 2021 | 264 | 614 | 43.00 | [38.98; 47.00] | 1.1% |
| Gluska et a., 2021 | 90 | 421 | 21.38 | [17.55; 25.60] | 1.1% |
| Harrison et a., 2021 | 123 | 251 | 49.00 | [42.67; 55.37] | 1.1% |
| Matsushima et a., 2021 | 160 | 558 | 28.67 | [24.99; 32.65] | 1.1% |
| Motrico et a., 2021 | 957 | 1954 | 48.98 | [46.73; 51.22] | 1.1% |
| Terada et a., 2021 | 35 | 461 | 7.59 | [ 4.89; 9.99] | 1.1% |
| Tsuno et a., 2021 | 104 | 558 | 18.64 | [15.52; 22.14] | 1.1% |
| Alfayumi-Zeadna et a., 2022 | 165 | 421 | 39.19 | [34.44; 44.01] | 1.1% |
| Brik et a., 2022 | 151 | 467 | 32.33 | [28.12; 36.79] | 1.1% |
| Chang et a., 2022 | 954 | 3253 | 29.33 | [27.76; 30.92] | 1.1% |
| Chrzan-Dętkoś et a., 2022 | 1318 | 1747 | 75.44 | [73.47; 77.58] | 1.1% |
| Dol et a., 2022 | 59 | 331 | 17.82 | [13.93; 22.44] | 1.1% |
| Eberhard-Gran et a., 2022 | 1164 | 3642 | 31.96 | [30.41; 33.47] | 1.1% |
| Fernandes et a., 2022 | 373 | 977 | 38.18 | [35.03; 41.26] | 1.1% |
| Gluska et a., 2022 | 53 | 421 | 12.59 | [ 9.48; 16.06] | 1.1% |
| Gómez-Baya et a., 2022 | 957 | 1954 | 48.98 | [46.73; 51.22] | 1.1% |
| Hiiragi et a., 2022 | 38 | 279 | 13.62 | [ 9.77; 18.17] | 1.1% |
| Hübner et a., 2022 | 6 | 62 | 9.68 | [ 4.25; 20.30] | 1.0% |
| Kuipers et a., 2022 | 29 | 148 | 19.59 | [13.66; 26.99] | 1.1% |
| Lequertier et a., 2022 | 270 | 1419 | 19.03 | [17.02; 21.17] | 1.1% |
| Myers et a., 2022 | 77 | 162 | 47.53 | [39.67; 55.50] | 1.1% |
| Nicolás-López et a., 2022 | 13 | 51 | 25.49 | [14.59; 39.71] | 1.0% |
| Orkaby et a., 2022 | 30 | 175 | 17.14 | [12.01; 23.65] | 1.1% |
| Pereira et a., 2022 | 83 | 207 | 40.10 | [33.37; 47.12] | 1.1% |
| Righetti et a., 2022 | 26 | 98 | 26.53 | [18.38; 36.52] | 1.1% |
| Takubo et a., 2022 | 173 | 1095 | 15.80 | [13.70; 18.11] | 1.1% |
| Tsuno et a., 2022 | 173 | 600 | 28.83 | [25.24; 32.64] | 1.1% |
| Viaux-Savelon et a., 2022 | 27 | 164 | 16.46 | [11.31; 23.16] | 1.1% |
| Zhang et a., 2022 | 12 | 85 | 14.12 | [ 7.90; 23.61] | 1.1% |
| Birkelund et a., 2023 | 82 | 526 | 15.59 | [12.59; 18.97] | 1.1% |
| Boisvert et a., 2023 | 64 | 216 | 29.63 | [23.74; 36.26] | 1.1% |
| Ciolac L et a., 2023 | 466 | 860 | 54.19 | [50.79; 57.55] | 1.1% |
| Costa R et a., 2023 | 183 | 648 | 28.24 | [24.77; 31.85] | 1.1% |
| Fuente-Moreno et a., 2023 | 522 | 1781 | 29.31 | [27.21; 31.49] | 1.1% |
| Harrison et a., 2023 | 1102 | 4611 | 23.90 | [22.53; 25.02] | 1.1% |
| Kovacheva et a., 2023 | 392 | 1954 | 20.06 | [18.29; 21.89] | 1.1% |
| Orsolini et a., 2023 | 14 | 144 | 9.72 | [ 5.62; 15.93] | 1.1% |
| Tsoneva et a., 2023 | 7 | 116 | 6.03 | [ 2.83; 12.34] | 1.1% |
| Fan HSL et a., 2025 | 1813 | 3817 | 47.50 | [45.89; 49.09] | 1.1% |
| **Random effects model** | | **43827** | **29.25** | **[24.58; 34.15]** | **53.5%** |
| Heterogeneity: $I^2 = 99.1\%$, $\tau^2 = 0.0333$, $p = 0$ | | | | | |
| **GDI – GROUP 2** | | | | | |
| An et a., 2020 | 70 | 209 | 33.49 | [27.20; 40.36] | 1.1% |
| Liang et a., 2020 | 259 | 864 | 29.98 | [26.68; 32.97] | 1.1% |
| Silverman et a., 2020 | 64 | 516 | 12.40 | [ 9.13; 15.08] | 1.1% |
| Stojanov et a., 2020 | 16 | 108 | 14.81 | [ 8.37; 22.71] | 1.1% |
| Baran et a., 2021 | 52 | 130 | 40.00 | [31.51; 48.95] | 1.1% |
| Bo et a., 2021 | 108 | 391 | 27.62 | [23.13; 32.27] | 1.1% |
| Feinberg et a., 2021 | 156 | 2372 | 6.58 | [ 5.60; 7.64] | 1.1% |
| Gildner et a., 2021 | 103 | 971 | 10.61 | [ 8.66; 12.64] | 1.1% |
| Gustafsson et a., 2021 | 48 | 146 | 32.88 | [25.35; 41.14] | 1.1% |
| Lewkowitz et a., 2021 | 54 | 204 | 26.47 | [20.13; 32.84] | 1.1% |
| Thompson et a., 2021 | 92 | 232 | 39.66 | [33.29; 46.26] | 1.1% |
| Yakupova et a., 2021 | 722 | 1645 | 43.89 | [41.44; 46.31] | 1.1% |
| Akyıldız et a., 2022 | 256 | 670 | 38.21 | [34.38; 41.94] | 1.1% |
| Howard et a., 2022 | 323 | 593 | 54.47 | [50.36; 58.58] | 1.1% |
| Hu et a., 2022 | 6 | 82 | 7.32 | [ 2.83; 15.33] | 1.0% |
| Kokkinaki et a., 2022 | 6 | 132 | 4.55 | [ 1.96; 9.87] | 1.1% |
| Micha et a., 2022 | 44 | 330 | 13.33 | [ 9.94; 17.55] | 1.1% |
| Shuman et a., 2022 | 256 | 670 | 38.21 | [34.52; 42.01] | 1.1% |
| Taljan et a., 2022 | 48 | 645 | 7.44 | [ 5.54; 9.75] | 1.1% |
| Waschmann et a., 2022 | 92 | 504 | 18.25 | [14.99; 21.92] | 1.1% |
| Wu et a., 2022 | 22 | 301 | 7.31 | [ 4.51; 10.75] | 1.1% |
| Altendahl et a., 2023 | 50 | 243 | 20.58 | [15.75; 26.26] | 1.1% |
| Kabinowitz et a., 2023 | 16 | 83 | 19.28 | [11.77; 29.58] | 1.1% |
| Zhang et a., 2023 | 330 | 468 | 70.51 | [66.31; 74.84] | 1.1% |
| Wang et a., 2024 | 499 | 2462 | 20.27 | [18.58; 21.81] | 1.1% |
| **Random effects model** | | **14971** | **23.92** | **[17.64; 30.82]** | **27.9%** |
| Heterogeneity: $I^2 = 98.9\%$, $\tau^2 = 0.0378$, $p = 0$ | | | | | |
| **GDI – GROUP 3** | | | | | |
| Guvenc et a., 2021 | 72 | 212 | 33.96 | [27.68; 40.79] | 1.1% |
| Miranda et a., 2021 | 113 | 305 | 37.05 | [31.59; 42.73] | 1.1% |
| Suárez-Rico et a., 2021 | 115 | 293 | 39.25 | [33.65; 45.11] | 1.1% |
| Erten et a., 2022 | 31 | 178 | 17.42 | [12.32; 23.92] | 1.1% |
| Sangsawang et a., 2022 | 38 | 126 | 30.16 | [22.48; 39.04] | 1.1% |
| Chávez-Tostado M et a., 2023 | 159 | 586 | 27.13 | [23.41; 30.81] | 1.1% |
| Aksoy et a., 2025 | 81 | 226 | 35.84 | [29.67; 42.50] | 1.1% |
| Miranda et a., 2025 | 268 | 659 | 40.67 | [36.91; 44.54] | 1.1% |
| **Random effects model** | | **2585** | **32.64** | **[27.30; 38.21]** | **8.9%** |
| Heterogeneity: $I^2 = 87.7\%$, $\tau^2 = 0.0060$, $p < 0.0001$ | | | | | |
| **GDI – GROUP 4** | | | | | |
| Lorentz et a., 2020 | 20 | 50 | 40.00 | [26.59; 54.78] | 1.0% |
| de Mola et a., 2021 | 305 | 1042 | 29.27 | [26.53; 32.15] | 1.1% |
| Galletta et a., 2021 | 69 | 184 | 37.50 | [30.46; 44.92] | 1.1% |
| Santos et a., 2022 | 30 | 101 | 29.70 | [21.06; 39.63] | 1.1% |
| Diniz BP et a., 2023 | 37 | 127 | 29.13 | [21.57; 37.92] | 1.1% |
| **Random effects model** | | **1504** | **31.80** | **[27.77; 35.98]** | **5.4%** |
| Heterogeneity: $I^2 = 43\%$, $\tau^2 = 0.0010$, $p = 0.1352$ | | | | | |
| **GDI – GROUP 5** | | | | | |
| Tariq et a., 2021 | 21 | 84 | 25.00 | [16.57; 35.80] | 1.1% |
| Afshari et a., 2022 | 409 | 600 | 68.17 | [64.31; 71.94] | 1.1% |
| Kawoos et a., 2022 | 7 | 56 | 12.50 | [ 5.92; 24.52] | 1.0% |
| Sudhinaraset et a., 2022 | 408 | 1072 | 38.06 | [35.13; 41.04] | 1.1% |
| **Random effects model** | | **1812** | **35.41** | **[14.06; 60.33]** | **4.3%** |
| Heterogeneity: $I^2 = 98.5\%$, $\tau^2 = 0.0631$, $p < 0.0001$ | | | | | |
| **Random effects model** | | **64699** | **28.48** | **[25.14; 31.94]** | **100.0%** |

Heterogeneity: $I^2 = 99.0\%$, $\tau^2 = 0.0318$, $p = 0$

Test for subgroup differences: $\chi^2_4 = 4.97$, df = 4 ($p = 0.2906$)

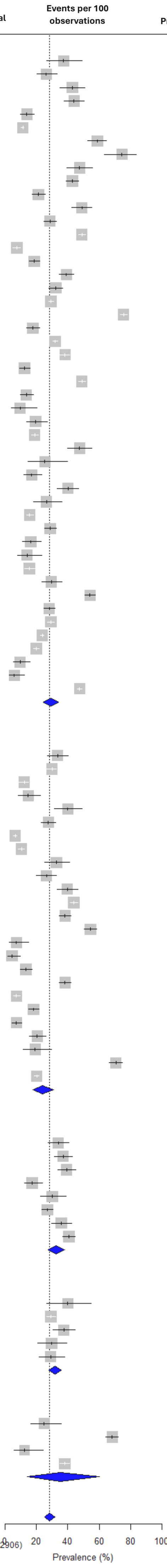

Supplement: Supplementary file 10 — Supplementary Material 10. [file 12884_2025_8262_MOESM10_ESM.pdf]
