# Supplementary material for: Prevalence of postpartum depression in the COVID-19 pandemic and associated factors: systematic review and meta-analysis
Source: BMC Pregnancy Childbirth. 2026 Jan 20;26:157. doi: 10.1186/s12884-025-08262-z (PMC12903221; doi:10.1186/s12884-025-08262-z)
Supplement: Supplementary file 11 — Supplementary Material 11: Methodological Quality Assessment of the Included Reviews According to the JBI Critical Appraisal for Analytical Cross-Sectional Studies. [25, 27, 28, 37, 47, 48, 52, 55, 57, 62, 66, 69, 73, 78, 79, 84–89, 91–94, 96, 97, 101, 102, 104, 105, 108, 112–116, 118, 119, 122, 124–126, 130, 170] [file 12884_2025_8262_MOESM11_ESM.pdf]

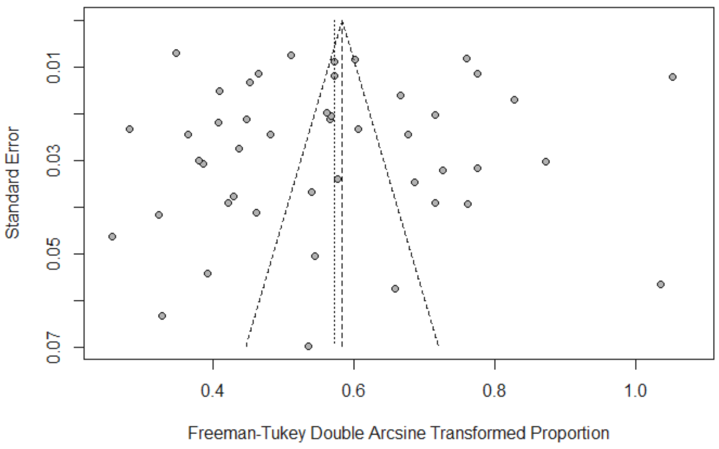

a)Funnel plot showing the prevalence of postpartum depression in Gender Inequality Index group 1 countries.

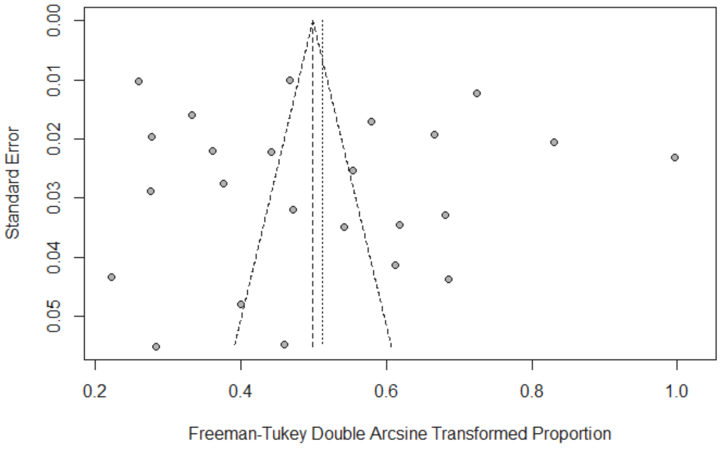

b)Funnel plot showing the prevalence of postpartum depression in Gender Inequality Index group 2 countries.

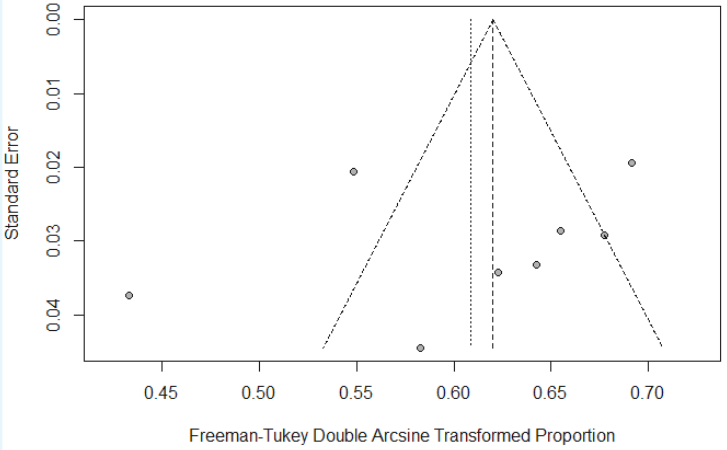

c)Funnel plot showing the prevalence of postpartum depression in Gender Inequality Index group 3 countries.

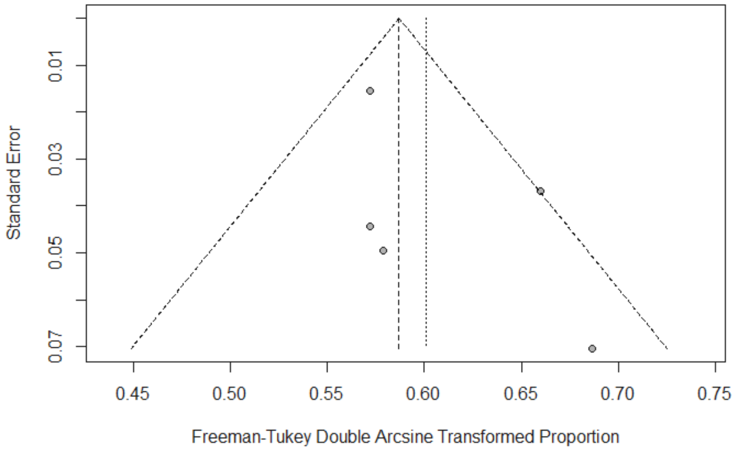

d)Funnel plot showing the prevalence of postpartum depression in Gender Inequality Index group 4 countries.

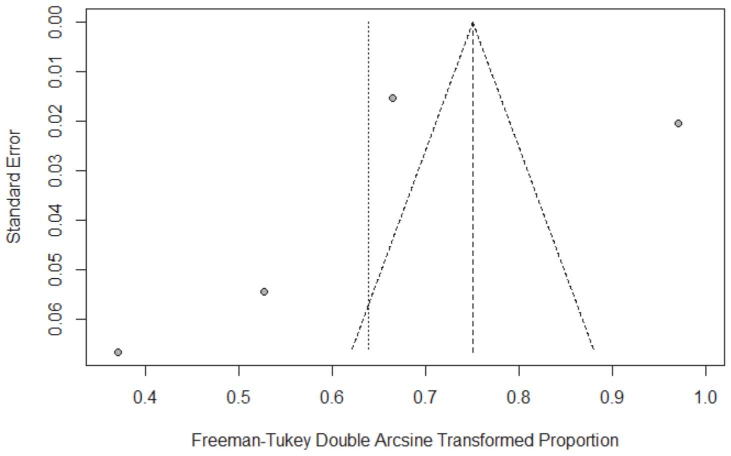

e)Funnel plot showing the prevalence of postpartum depression in Gender Inequality Index group 5 countries.
