# Supplementary material for: Prevalence of postpartum depression in the COVID-19 pandemic and associated factors: systematic review and meta-analysis
Source: BMC Pregnancy Childbirth. 2026 Jan 20;26:157. doi: 10.1186/s12884-025-08262-z (PMC12903221; doi:10.1186/s12884-025-08262-z)

Study	Events	Total	Prevalence	95%-CI	Weight
**Postpartum = up to 1 month**					
Mariño-Narvaez et a., 2020	28	75	37.33	[26.71; 49.28]	1.2%
Gildner et a., 2021	103	971	10.61	[7.98; 12.03]	1.3%
Terada et a., 2021	35	461	7.59	[4.87; 9.97]	1.3%
Hiiragi et a., 2022	38	279	13.62	[9.75; 18.16]	1.3%
Nicolás-López et a., 2022	13	51	25.49	[14.79; 39.77]	1.1%
Santos et a., 2022	30	101	29.70	[21.18; 39.67]	1.2%
Takubo et a., 2022	173	1095	15.80	[13.39; 17.83]	1.3%
Kabinowitz et a., 2023	16	83	19.28	[11.75; 29.58]	1.2%
**Random effects model**		**3116**	**18.31**	**[11.90; 25.70]**	**10.0%**
Heterogeneity: $I^2 = 90.9\%$, $\tau^2 = 0.0145$, $p < 0.0001$					
**Postpartum = up to 3 months**					
Liang et a., 2020	259	864	29.98	[26.96; 33.17]	1.3%
Ceulemans et a., 2021	592	5134	11.53	[10.53; 12.31]	1.3%
Fallon et a., 2021	264	614	43.00	[39.05; 47.02]	1.3%
Feinberg et a., 2021	156	2372	6.58	[5.36; 7.41]	1.3%
Galletta et a., 2021	69	184	37.50	[30.56; 44.94]	1.3%
Guvenc et a., 2021	72	212	33.96	[27.71; 40.80]	1.3%
Suárez-Rico et a., 2021	115	293	39.25	[33.59; 45.09]	1.3%
Erten et a., 2022	31	178	17.42	[12.32; 23.92]	1.3%
Gluska et a., 2022	53	421	12.59	[9.40; 15.99]	1.3%
Hu et a., 2022	6	82	7.32	[3.06; 15.50]	1.2%
Kokkinaki et a., 2022	6	132	4.55	[1.85; 9.77]	1.2%
Micha et a., 2022	44	330	13.33	[9.78; 17.42]	1.3%
Orkaby et a., 2022	30	175	17.14	[11.97; 23.62]	1.3%
Pereira et a., 2022	83	207	40.10	[33.44; 47.13]	1.3%
Righetti et a., 2022	26	98	26.53	[18.33; 36.50]	1.2%
Sangsawang et a., 2022	38	126	30.16	[22.46; 39.03]	1.2%
Shuman et a., 2022	256	670	38.21	[34.47; 41.99]	1.3%
Taljan et a., 2022	48	645	7.44	[5.53; 9.74]	1.3%
Viaux-Savelon et a., 2022	27	164	16.46	[11.35; 23.19]	1.3%
Waschmann et a., 2022	92	504	18.25	[14.99; 21.92]	1.3%
Zhang et a., 2022	12	85	14.12	[7.93; 23.63]	1.2%
Altendahl et a., 2023	50	243	20.58	[15.73; 26.25]	1.3%
Birkelund et a., 2023	82	526	15.59	[12.65; 19.03]	1.3%
Boisvert et a., 2023	64	216	29.63	[23.73; 36.26]	1.3%
Chávez-Tostado M et a., 2023	159	586	27.13	[23.54; 30.90]	1.3%
Diniz BP et a., 2023	37	127	29.13	[21.63; 37.95]	1.2%
Tsoneva et a., 2023	7	116	6.03	[2.59; 12.15]	1.2%
Wang et a., 2024	499	2462	20.27	[18.68; 21.89]	1.3%
**Random effects model**		**17766**	**20.78**	**[16.52; 25.38]**	**35.8%**
Heterogeneity: $I^2 = 97.8\%$, $\tau^2 = 0.0204$, $p < 0.0001$					
**Postpartum = up to 6 months**					
Lorentz et a., 2020	20	50	40.00	[26.81; 54.74]	1.1%
Molgora et a., 2020	49	186	26.34	[20.31; 33.37]	1.3%
Boudiaf et a., 2021	37	264	14.02	[10.07; 18.80]	1.3%
Chaves et a., 2021	161	274	58.76	[52.67; 64.62]	1.3%
Emmott et a., 2021	77	162	47.53	[39.58; 55.55]	1.3%
Motrico et a., 2021	957	1954	48.98	[46.71; 51.23]	1.3%
Yakupova et a., 2021	722	1645	43.89	[41.42; 46.31]	1.3%
Afshari et a., 2022	409	600	68.17	[64.26; 71.86]	1.3%
Akyıldız et a., 2022	256	670	38.21	[34.49; 42.00]	1.3%
Chang et a., 2022	954	3253	29.33	[27.64; 30.81]	1.3%
Dol et a., 2022	59	331	17.82	[13.27; 21.93]	1.3%
Fernandes et a., 2022	373	977	38.18	[35.05; 41.27]	1.3%
Gómez-Baya et a., 2022	957	1954	48.98	[46.72; 51.22]	1.3%
Myers et a., 2022	77	162	47.53	[39.60; 55.53]	1.3%
Wu et a., 2022	22	301	7.31	[4.43; 10.68]	1.3%
Costa R et a., 2023	183	648	28.24	[24.59; 31.73]	1.3%
Fuente-Moreno et a., 2023	522	1781	29.31	[27.18; 31.47]	1.3%
Harrison et a., 2023	1102	4611	23.90	[22.65; 25.14]	1.3%
Kovacheva et a., 2023	392	1954	20.06	[18.29; 21.90]	1.3%
**Random effects model**		**21777**	**34.67**	**[27.37; 42.35]**	**24.6%**
Heterogeneity: $I^2 = 98.8\%$, $\tau^2 = 0.0299$, $p < 0.0001$					
**Postpartum = up to 12 months**					
Ostacoli et a., 2020	70	163	42.94	[35.30; 50.92]	1.3%
Spinola et a., 2020	107	243	44.03	[37.72; 50.52]	1.3%
Stojanov et a., 2020	16	108	14.81	[8.84; 23.02]	1.2%
de Mola et a., 2021	305	1042	29.27	[26.50; 32.12]	1.3%
Gustafsson et a., 2021	48	146	32.88	[25.42; 41.16]	1.3%
Harrison et a., 2021	123	251	49.00	[42.61; 55.41]	1.3%
Lewkowitz et a., 2021	54	204	26.47	[20.58; 33.10]	1.3%
Miranda et a., 2021	113	305	37.05	[31.64; 42.75]	1.3%
Thompson et a., 2021	92	232	39.66	[33.31; 46.26]	1.3%
Tsuno et a., 2021	104	558	18.64	[15.49; 22.12]	1.3%
Alfayumi-Zeadna et a., 2022	165	421	39.19	[34.52; 44.04]	1.3%
Chrzan-Dętkoś et a., 2022	1318	1747	75.44	[73.38; 77.47]	1.3%
Eberhard-Gran et a., 2022	1164	3642	31.96	[30.30; 33.38]	1.3%
Howard et a., 2022	323	593	54.47	[50.36; 58.52]	1.3%
Kuipers et a., 2022	29	148	19.59	[13.77; 27.06]	1.3%
Lequertier et a., 2022	270	1419	19.03	[16.65; 20.84]	1.3%
Sudhinaraset et a., 2022	408	1072	38.06	[35.15; 41.05]	1.3%
Tsuno et a., 2022	173	600	28.83	[25.11; 32.55]	1.3%
Ciolac L et a., 2023	466	860	54.19	[50.79; 57.61]	1.3%
Orsolini et a., 2023	14	144	9.72	[5.63; 15.94]	1.3%
Zhang et a., 2023	330	468	70.51	[66.13; 74.57]	1.3%
Aksoy et a., 2025	81	226	35.84	[29.59; 42.47]	1.3%
Miranda et a., 2025	268	659	40.67	[36.89; 44.53]	1.3%
**Random effects model**		**15251**	**36.55**	**[29.72; 43.66]**	**29.7%**
Heterogeneity: $I^2 = 98.9\%$, $\tau^2 = 0.0306$, $p = 0$					
**Random effects model**		**57910**	**28.33**	**[24.76; 32.04]**	**100.0%**
Heterogeneity: $I^2 = 99.0\%$, $\tau^2 = 0.0319$, $p = 0$					
Test for subgroup differences: $\chi^2_3 = 23.75$, df = 3 ($p < 0.0001$)					

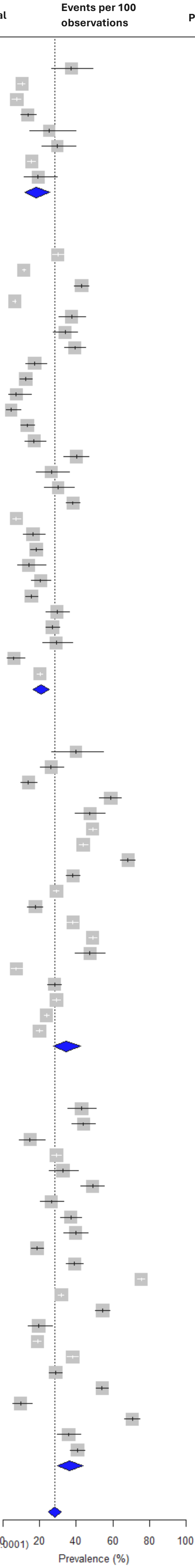

Supplement: Supplementary file 12 — Supplementary Material 12: Table with description of articles included in the meta-analysis. [25, 27, 28, 37, 47, 48, 52, 55, 57, 62, 66, 67, 69, 73, 75, 78–80, 84–94, 96, 97, 102–106, 108, 112–116, 118, 119, 122, 124–126, 130, 170] [file 12884_2025_8262_MOESM12_ESM.pdf]
