# Supplementary material for: Prevalence of postpartum depression in the COVID-19 pandemic and associated factors: systematic review and meta-analysis
Source: BMC Pregnancy Childbirth. 2026 Jan 20;26:157. doi: 10.1186/s12884-025-08262-z (PMC12903221; doi:10.1186/s12884-025-08262-z)
Supplement: Supplementary file 13 — Supplementary Material 13: Table with risk and protective factors for postpartum depression during the COVID-19 pandemic. [25, 27, 28, 37, 47, 48, 52, 55, 57, 62, 66, 69, 75, 79, 80, 84, 85, 87–89, 91–94, 96, 97, 102–104, 108, 112, 115, 116, 118, 119, 122, 124–126, 130, 170] [file 12884_2025_8262_MOESM13_ESM.pdf]

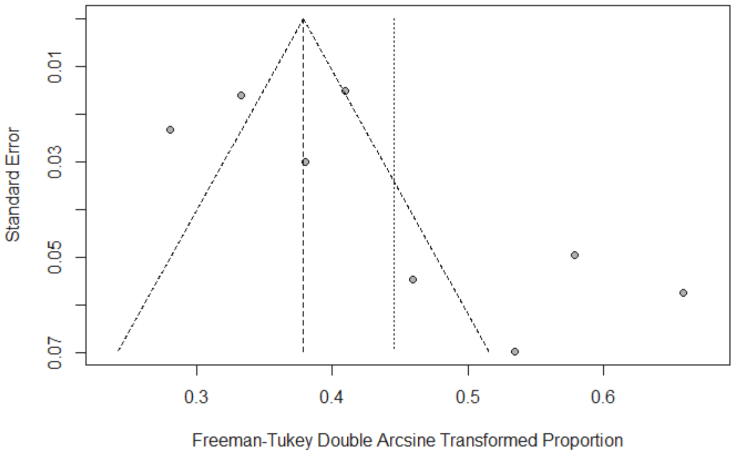

a)Funnel plot with prevalence of postpartum depression up to 1 month after birth.

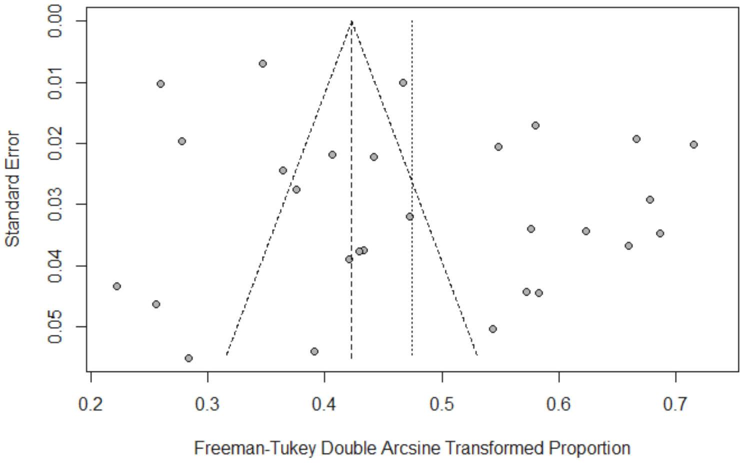

b)Funnel plot with prevalence of postpartum depression up to 3 months after birth.

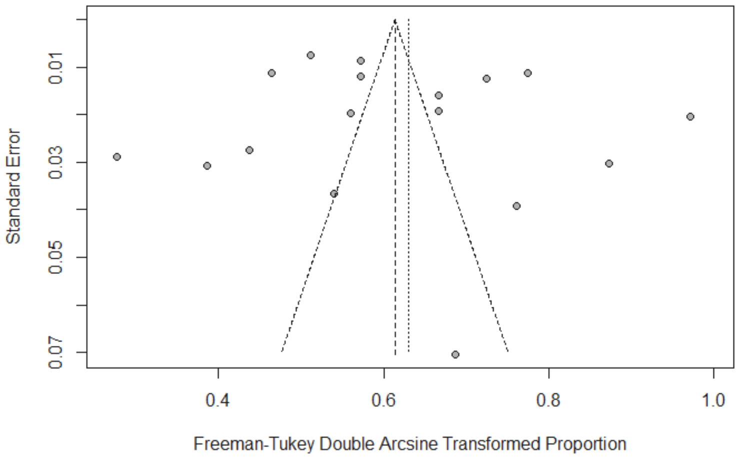

c)Funnel plot with prevalence of postpartum depression up to 6 months after birth.

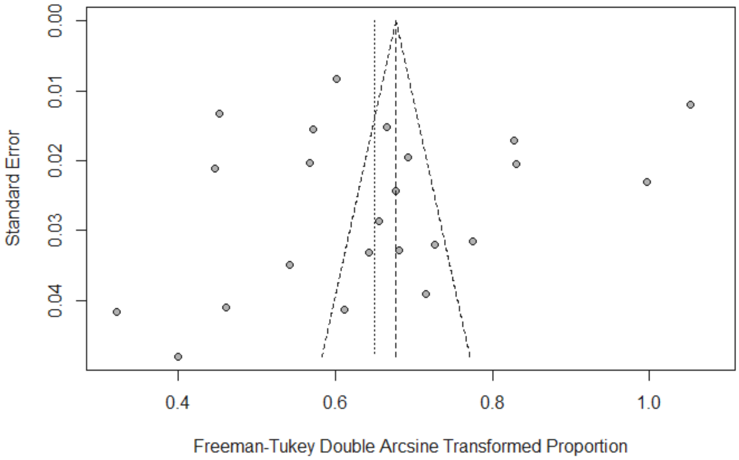

d)Funnel plot with prevalence of postpartum depression up to 12 months after birth.
